# Cytochemical localization and synthesis mechanism of the glucomannan in pseudobulbs of *Bletilla striata* Reichb. f

**DOI:** 10.1093/hr/uhae092

**Published:** 2024-04-22

**Authors:** Junfeng Huang, Shuang Ma, Ming Zhou, Zhihao Liu, Qiong Liang

**Affiliations:** Key Laboratory of Plant Germplasm Enhancement and Specialty Agriculture, Wuhan Botanical Garden, Chinese Academy of Sciences, Wuhan 430074, China; Key Laboratory of Plant Germplasm Enhancement and Specialty Agriculture, Wuhan Botanical Garden, Chinese Academy of Sciences, Wuhan 430074, China; Key Laboratory of Plant Germplasm Enhancement and Specialty Agriculture, Wuhan Botanical Garden, Chinese Academy of Sciences, Wuhan 430074, China; Key Laboratory of Edible Wild Plants Conservation and Utilization, Hubei Normal University, Huangshi City 435002, China; Key Laboratory of Plant Germplasm Enhancement and Specialty Agriculture, Wuhan Botanical Garden, Chinese Academy of Sciences, Wuhan 430074, China; Institute of Hydrobiology, Chinese Academy of Sciences, Wuhan 430072, China

## Abstract

The dried pseudobulbs of *Bletilla striata*, an important traditional Chinese medicine named *BaiJi*, have an extraordinary polysaccharide content and excellent prospects for medicinal effects. However, the distribution and molecular mechanism underlying biosynthesis are poorly understood. In this study, chemical and immunologic analyses were performed in representative tissues of *B. striata*, and the results showed that what are conventionally termed *Bletilla striata* polysaccharides (BSPs) are water-soluble polysaccharides deposited only in pseudobulbs. The structural component of BSPs is glucomannan, with a mannose:glucose mass ratio of ~3:2. BSPs are present in the parenchyma of the pseudobulbs in cells known as glucomannan idioblasts and distributed in the cytoplasm within cellular membranes, but are not contained in the vacuole. Comparative transcriptomics and bioinformatics analyses mapped the pathway from sucrose to BSP and identified *BsGPI*, *BsmanA*, and *BsCSLA*s as the key genes of BSP biosynthesis, suggesting that the functional differentiation of the cellulose synthase-like family A (CSLA) may be critical for the flow of glucomannan to the BSP or cell wall. Subsequently, virus-mediated gene silencing showed that silencing of two CSLAs (*Bs03G11846* and *Bs03G11849*) led to a decrease in BSP content, and yeast two-hybrid and luciferase complementation experiments confirmed that four CSLAs (Bs03G11846, Bs03G11847, Bs03G11848, and Bs03G11849) can form homo- or heterodimers, suggesting that multiple CSLAs may form a large complex that functions in BSP synthesis. Our results provide cytological evidence of BSP and describe the isolation and characterization of candidate genes involved in BSP synthesis, laying a solid foundation for further research on its regulation mechanisms and the genetic engineering breeding of *B. striata*.

## Introduction

The dry pseudobulb of the *Bletilla striata* plant, named *BaiJi* in Chinese as an important traditional Chinese medicine (TCM), has the effects of astringent hemostasis, detumescence, and promotion of muscle growth. It was firstly recorded in the Chinese medical classic ‘Shennong’s herb’ [1]. There are six species in the *Bletilla* (Orchidaceae) genus, distributed in Asia from northern Myanmar, China, to Japan, with high medicinal and ornamental values [2], and four species in China, including *B. striata* Rchb. f., *B. formosana* Schltr., *B. ochracea* Schltr., and *B. sinensis* Schltr. (http://www.iplant.cn/info/Bletilla?t=foc). *Bletilla striata* is a perennial herb with one inflorescence stem, four to six leaves enclosing the inflorescence stem to form the shoot, and an underground stem in the form of a corm (also known as a pseudobulb or tuber). The leaf sheaths and pedicel form the stem, which grows directly on the fleshy pseudobulb, a triangular-shaped, flat, spherical or irregular rhomboidal structure. The shoots wither in winter, and about two to four new buds emerge from the pseudobulb to form new daughter plants in the following spring. Perennial pseudobulbs, as dormant corms, no longer have shoots and are connected in clusters.

A soluble, non-cellulosic polysaccharide is the key active ingredient of *B. striata* (*Bletilla striata* polysaccharide, BSP), which is widely used in clinical hemostasis and can also be used as an excellent biopolymer material and pharmaceutical excipient [3–5]. The chemical composition of BSP is glucomannan, which has a linear backbone consisting mainly of repeated β-1,4-linked d-mannosyl residues and β-1,4-linked d-glucosyl residues, and its content in the dry pseudobulb varies from 8.52 to 59.77% [6–8]. The molar ratio of mannose (Man) to glucose (Glc) in BSP is controversial, with 1.6:1, 3:1, 3:2, and 3.76:1 being reported [9, 10]. Glucomannan is a heteromannan (HM), which, together with homomannan, belongs to the mannan polymers that accumulate as storage polysaccharides in specialized tissues for health-promoting food and medicinal uses [11, 12], and is also a hemicellulose component of the lignified cell walls that serves as a structural polymer [13, 14]. However, the cellular distribution of glucomannan in *B. striata* is unknown.

In general, the synthesis of the soluble polysaccharide but not the cell wall polysaccharide is poorly understood. A series of enzymes are required to convert the sucrose produced by photosynthesis into Glc and Man and polymerize them into glucomannan of the cell wall [15]. The first phase of the pathway is sucrose (SUC) transport, which transports photosynthesis-produced sucrose from the source to sink tissues [16]. The second phase is the synthesis of monosaccharide substrate-active Man and Glc. Firstly, sucrose is hydrolyzed into monosaccharide d-fructose (Fru) and d-Glc or UDP-Glc by cell wall invertases (CWINVs) or sucrose synthase (SUS), which are encoded by *sacA* and *SUS*, respectively [17, 18]. Secondly, d-Fru is converted to GDP-Man, the widely accepted monosaccharide substrate of mannan and HM:glucomannan, galactomannan (GM), and galactoglucomannan (GGM), under the action of fructokinase (*scrK*), Man-6-phosphate isomerase (*manA*), phosphomannomutase (*PMM*), and Man-1-phosphate guanylyltransferase (*GMPP*) [7, 19]. However, there are still many controversies and uncertainties about Glc, another monosaccharide substrate for glucomannan and GM. The *in vitro* synthesis of glucomannan depends on the presence of GDP-Glc and cannot be replaced with UDP-Glc, the precursor for cellulose synthesis and other ß-1,4-glucans [15, 20]. UDP-Glc is the most abundant nucleotide sugar in plants, and there is no obvious spike in GDP-Glc in *Arabidopsis* and tobacco [21, 22]. It remains unclear how GDP-Glc is generated in plant cells, as it still seems to be missing the GDP-Glc pyrophosphorylase. d-Glc is catalyzed to UDP-Glc by hexokinase, phosphoglucomutase, and uridine-diphosphate Glc pyrophosphorylase, which are encoded by *HK*, *pgm*, and *ugp2*, respectively [23]. Glc-6-phosphate isomerase (GPI) catalyzes the interconversion of d-Glc-6-phosphate and d-Fru-6-phosphate [24]. In the last phase, the β-1,4-glucomannan backbone is catalyzed by Golgi-localized mannan synthases, which are members of the cellulose synthase-like family A (CSLA) of glycosyltransferase family A [14, 25, 26]. Two mannan synthesis-related (MSR) proteins are also involved in glucomannan biosynthesis because the *Arabidopsis msr1msr2* double mutant led to a reduction in mannosyl residue levels in stem glucomannan [27], and AtMSR1 is an enhancer of AtCSLA2, which can co-catalyze glucomannan synthesis, whereas AtCSLA2 alone can only synthesize mannan in yeast [28]. The small intracellular punctae-localized *Amorphophallus konjac* CSLA3 expression alone in Pichia cells is sufficient to produce glucomannan in *Pichia* cells.

Research on *B. striata* mainly focuses on chemical components, pharmacological activities, and reproductive techniques [29–31]. Metabolomics, transcriptomics, and genomics started later but are proceeding at a rapid pace [1, 32, 33]. A large-scale unigene database and set of EST-SSRs had been generated from multiple tissues, not including the pseudobulb of *B. striata*, based on RNA-seq [34, 35]. Furthermore, eight enzyme genes related to monosaccharide substrate biosynthesis in the polysaccharide pathway were identified by *de novo* RNA sequencing [7]. But so far only some enzyme genes have been cloned from *B. striata*, including the GDP-mannose pyrophosphorylase gene *GMP*, the mannose synthesis gene *phosphomannomutase* (*PMM*) [36], the phenylalanine ammonia-lyase gene *PAL* [37], and the Cu–Zn superoxide dismutase (SOD, *BsSOD1* and *BsSOD2*) [38] by RACE–PCR. A complete and full-length transcriptome of *B. striata* was obtained by third-generation single-molecule real-time (SMRT) sequencing and next-generation RNA-sequencing (NGS) using mature capsule-induced callus [33]. However, the RNA-seq data of different stages of the pseudobulb, the medicinal tissue of *B. striata*, is absent, which may mask useful information related to the biosynthetic pathway of the active ingredients. More recently, two high-quality haplotype-resolved genomes of *B. striata* have been assembled, and an MYB transcriptional repressor, BsMYB1, has been identified to regulate BSP biosynthesis by interacting with nine enzyme gene families [1]. However, there is no evidence that these enzymes are involved in BSP synthesis.

Hence, we analyzed the content, composition, and distribution of BSP, obtained candidate enzyme genes involved in BSP biosynthesis, and characterized the CSLA gene family in the glucomannan polymerization of the cell wall and BSP. The results provide a valuable resource for future elucidation of molecular mechanisms in the medicinal active ingredients of *B. striata*.

## Results

### *Bletilla striata* polysaccharides are glucomannan with a mannose:glucose ratio of approximately 3:2

To clarify the composition of BSPs, we investigated the qualitative and quantitative differences of polysaccharides in different tissues of *B. striata*. Firstly, we determined the water-soluble polysaccharide (water extraction and alcoholic precipitation method) content by the weighing method and the polysaccharide content by the phenol–sulfuric acid method using glucose as the standard in various tissues, including roots, stems, leaves, and 1-, 2-, 3-, and 4-year-old pseudobulbs (1P, 2P, 3P, and 4P), and the results showed that the water-soluble polysaccharide content in pseudobulb tissues (1P, 2P, 3P, and 4P) was much higher than that in non-pseudobulb tissues (roots, stems, and leaves). Meanwhile, it is worth noting that the content of polysaccharides in pseudobulbs sharply decreased with increasing growth years, and was as high as 61.50% in 1P (Table 1). Next, we determined the monosaccharide composition of water-soluble polysaccharides extracted from different tissues by gas chromatography (GC) using a two-step silylation derivatization procedure, and the results showed that there were differences in the composition and ratio of monosaccharides in pseudobulb tissues and non-pseudobulb tissues. Specifically, the water-soluble polysaccharide in pseudobulb tissues was composed of Man and Glc with a mass ratio of 1.69:1, 1.54:1, 1.38:1, and 1.44:1 in 1P, 2P, 3P, and 4P, respectively. In non-pseudobulb tissues, the water-soluble polysaccharides of roots and leaves had a certain amount of galactose (Gal) and arabinose (Ara) in addition to Man and Glc, with ratios of 4.40:1 and 5. 59:1, respectively, and a small amount of three unknown monosaccharide residues, while the content of unknown 2 in leaves was high. The water-soluble polysaccharides could hardly be extracted from the stems, and there were no signals of xylose and rhamnose in all samples (Table 1; Supplementary Data Figs S2 and S3; Supplementary Data Table S1). These results indicate that what are conventionally called BSPs were the water-soluble polysaccharides only isolated from the pseudobulbs of *B. striata*, and BSPs were glucomannan with a mass ratio of ~3:2 (Man:Glc), in agreement with previous results [6, 10].

**Table 1:** Polysaccharide content and monosaccharide composition in various tissues of *B. striata*

**Tissue**	**Water-soluble**	**Monosaccharide composition**				**Polysaccharide**
	**polysaccharide content (%)**	Mannose	Glucose	Galactose	Arabinose	**content (%)**
Root	5.59 ± 0.22^e^	189.09 ± 8.73	42.99 ± 1.99	18.52 ± 0.86	4.73 ± 0.63	0.71 ± 0.08^d^
Stem	1.96 ± 0.15^f^	ns	ns	ns	ns	0.57 ± 0.03^d^
Leaf	4.10 ± 0.05^e^	18.52 ± 0.65	3.31 ± 0.12	3.52 ± 0.12	3.67 ± 0.72	0.67 ± 0.03^d^
1P	49.08 ± 1.55^a^	374.00 ± 12.35	211.62 ± 13.70	ns	ns	61.50 ± 8.63^a^
2P	39.49 ± 3.16^b^	324.78 ± 18.96	210.10 ± 7.45	ns	ns	38.20 ± 7.73^b^
3P	25.94 ± 1.40^c^	418.38 ± 21.63	304.40 ± 10.54	ns	ns	25.70 ± 3.87^c^
4P	14.16 ± 1.03^d^	344.13 ± 13.26	239.23 ± 9.08	ns	ns	6.38 ± 0.37^d^

Data shown are mean ± standard deviation (*n* = 3) and different lowercase letters with superscripts indicate significant differences with the parameter of *P* < 0.05. ns, no signal. There are no signals of xylose and rhamnose in all samples.

### *Bletilla striata* polysaccharides are distributed in the cytoplasm of glucomannan idioblasts in pseudobulbs

**Figure 1:**
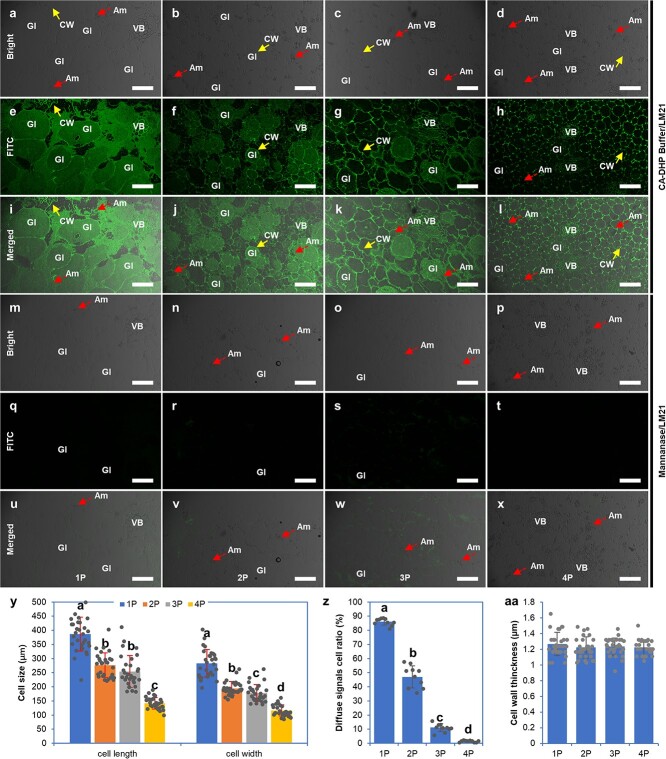
Immunolocalization of glucomannan labeled by monoclonal antibody LM21 in four developmental stages of pseudobulbs of *B. striata.* In sections pretreated with β-mannanase dissolved in citric acid–disodium hydrogen phosphate (CA-DHP) buffer for 4 h, the FITC fluorescence signals disappeared (**m**–**x**). Sections pretreated with CA-DHP buffer (**a**–**l**) have strong FITC fluorescence signals in the cell wall (CW, solid arrows) and cytoplasm in glucomannan idioblasts (GIs). **y** The size of pseudobulb cells at different developmental stages was represented by cell length and cell width. **z** Proportion of cells with diffuse signals in the whole cell to the total cell number. **aa** Cell wall thickness was analyzed by ImageJ using micrographs in a bright field. Values represent the mean ± standard deviation (*n* = 10 in **z**, *n* = 30 in **y** and **aa**). Different lowercase letters represent significant differences with a *P* < 0.05 cutoff. Am, amyloplast (dashed arrows); VB, vascular bundle. Scale bars = 200 μm.

To detect the distribution of BSP in pseudobulb cells using immunohistochemistry (IHC) and immunocolloidal gold (ICG) assays, roots, stems, leaves, and 1P, 2P, 3P, and 4P were sampled for paraffin-embedded and subsequent IHC. After clarifying that the BSP structure was glucomannan, we first used the periodic acid–Schiff (PAS) reagent to display the BSP [12]. However, the results showed that the PAS reagent could not be used to color the BSP (Supplementary Data Fig. S4). LM21, an antibody that could specifically recognize β-linked mannan polysaccharides, was used to label the BSP. There are many strong signals diffusely distributed within the egg-shaped idioblasts (glucomannan idioblasts, GIs) within the parenchyma of pseudobulbs (Fig. 1a–l), and the signal was lost when mannanase was added (Fig. 1m–x). The fluorescence signal of BSP could be significantly distinguished from the fluorescein isothiocyanate (FITC) fluorescence signal of the cell wall, which contains mannans, glucomannan, and galactomannan polysaccharides, which can also be bound by the LM21 primary antibody and disappeared on mannanase hydrolysis (Fig. 1a–x). The intracellular diffuse signals were present only in pseudobulbs, and the signals in roots, stems, and leaves were only present in the cell wall (Supplementary Data Fig. S5). With the progression of pseudobulb development, the fluorescence intensity of the diffuse signal (Fig. 1e–h), pseudobulb cell size (Fig. 1y), and the ratio of cells with diffuse signals to the cells in the view all decreased (Fig. 1z). Nevertheless, the cell wall thickness of GIs in the pseudobulbs did not show differences (Fig. 1aa), which suggested that the BSP in the pseudobulbs was cytoplasmic glucomannan rather than a cell wall component.

**Figure 2:**
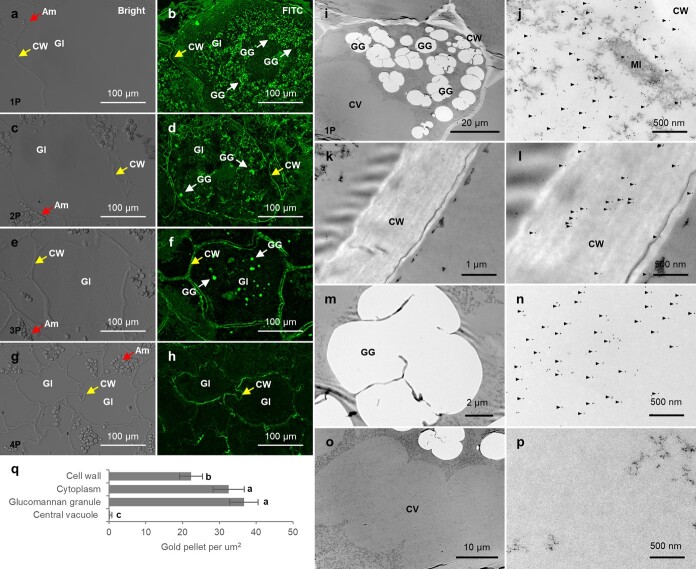
Localization of glucomannan recognized by the LM21 monoclonal antibody in glucomannan idioblasts of *B. striata* pseudobulb using IHC and ICG. **a**–**h** IHC reveals the distribution of glucomannan in pseudobulb GIs at four developmental stages. The left column represents the bright-field view under confocal microscopy, and the right column represents the FITC fluorescence field view. Scale bars = 100 μm. **i**–**p** TEM micrographs using ICG show localization of glucomannan within the GIs of 1P. **i** Full view of each microstructure. **j** Cytoplasm. **k**, **l** Cell wall outline (**k**) and an enlarged view of the cell wall sufficient to show the gold pellet signal (**l**). **m**, **n** Glucomannan granule. **o**, **p** Central vacuole. The scale bars are as follows: 20 μm (**i**), 1 μm (**k**), 2 μm (**m**), 10 μm (**o**), and 500 nm (**j**, **l**, **n**, and **p**). **q** Density of gold pellet signal. Values are mean ± standard deviation (*n* ≥ 6). Different lowercase letters represent significant differences with a *P* < 0.05 cutoff. CW, cell wall; Am, amyloplast; GG, glucomannan granule; CV, central vacuole; MI, mitochondrion.

The field of view under higher-resolution conditions revealed that the diffuse glucomannan signal was strongest in the GIs of 1P (Fig. 2a and b), and the diffuse-like signal decreased gradually as development progressed, aggregating into granules, and gradually becoming larger, which was more evident in 2P and 3P (Fig. 2c–f). In 4P the intracellular glucomannan signal almost disappeared, as in other non-pseudobulb tissues (Fig. 2g and h), which was consistent with the polysaccharide content in pseudobulbs at different growth stages. Since glucomannan diffusivity and particle distribution were most abundant in 1P, ICG was performed to present more details of BSP distribution. Transmission electron microscopy (TEM) results showed that gold pellets identifying LM21 appeared in the cell wall and the cytoplasmic region excluding the vacuoles, the latter having a higher density of gold pellets than the former (Fig. 2i–q). The signal in the cytoplasm was the same as the IHC results, mainly diffused or aggregated into granules (Fig. 2j and n).

### Comparative transcriptomic simplicity reveals the molecular mechanism of the high polysaccharide content in pseudobulbs

To dissect the molecular mechanism of the high water-soluble polysaccharide content in the pseudobulbs, we performed comparative RNA-seq analysis between pseudobulb tissues (1P, 2P, 3P, and 4P), non-pseudobulb tissues (roots, stems, leaves, and flowers), and transitional stages (seedling, containing roots, pseudobulbs, stems, and leaves). Considering the high polysaccharide content in pseudobulbs, especially in 1P, multiple tissues with 1P were grouped to compare differentially expressed genes (DEGs). There were more DEGs between pseudobulb tissue and non-pseudobulb tissue and fewer DEGs between pseudobulb tissues at different developmental stages (Supplementary Data Fig. S6). The hierarchical cluster result of DEGs among different samples showed that the above-ground parts (buds, leaves, stems, and flowers) form one category, and the underground parts, including roots, pseudobulbs of different growth years, and seedlings, form another category. Simultaneously, we found that 1P and 2P were divided into a subcategory, whereas 3P and 4P were divided into another separate subcategory (Supplementary Data Fig. S7).

The DEGs in different groups were individually analyzed by KEGG, and the results showed that starch and sucrose metabolism (map00500), plant hormone signal transduction (map04075), MAPK signaling pathway-plant (map04016), fructose and mannose metabolism (map00051), and stilbenoid, diarylheptanoid and gingerol biosynthesis (map00945) pathways were significantly enriched (Supplementary Data Table S2). Therefore, we further annotated the DEGs matched to the pathways of map00051 and map00500, which are responsible for the conversion of sucrose into monosaccharide substrates for polysaccharide synthesis. The results showed that, on the one hand, genes that encode the enzymes responsible for converting sucrose into UDP- or GDP-monosaccharide, which are the substrates for polysaccharide synthesis, were upregulated in 1-year-old pseudobulb compared with non-pseudobulb tissues, such as *INV*, *HK*, *manA*, *PMM*, and *pgm*. On the other hand, genes that encode E3.2.1.4 (endoglucanase), E3.2.1.21 (β-glucosidase), and E3.2.1.39 (glucan endo-1,3-β-d-glucosidase), which are the candidate enzymes responsible for the hydrolysis of polysaccharides (cellulose, β-d-glucoside, and 1,3-β-glucan) to monosaccharides, were down-regulated in 1P compared with non-pseudobulb tissues (Supplementary Data Figs S8 and S9). These results provide a molecular basis for the high polysaccharide content in the pseudobulb and the metabolic flux for the biosynthesis of BSP.

### Identification and characterization of *B. striata* polysaccharide-related genes

**Figure 3:**
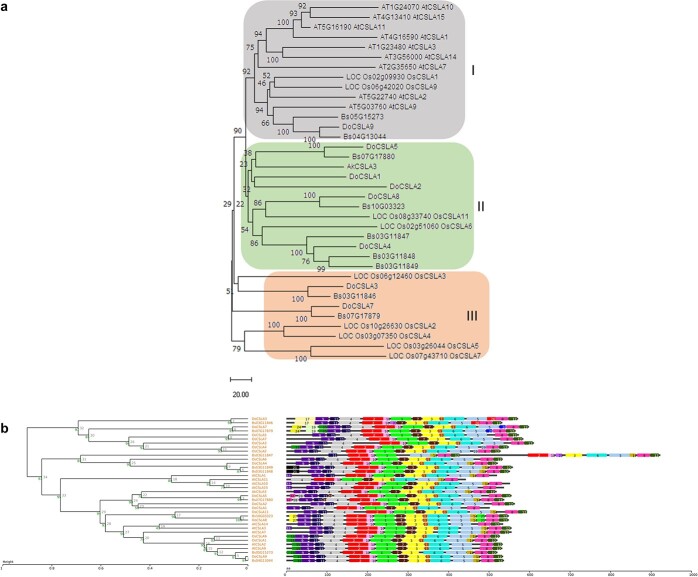
Phylogenetic relationship of nine *B. striata* (Bs) CSLA homologs, nine *Arabidopsis* (At), nine *Oryza sativa* (Os), eight *Dendrobium officinale* (Do), and one *Amorphophallus konjac* (Ak) CSLA members. **a** Phylogenetic analysis of the CSLA family of *B. striata*, *A. thaliana* from TAIR, *O. sativa* from the China Rice Data Center (www.ricedata.com), and *D. officinale*, and *A. konjac* from NCBI by MEGA11 with the same method as Supplementary Data Fig. S10. AtCSLA1 (AT4G16590), AtCSLA2 (AT5G22740), AtCSLA3 (AT1G23480), AtCSLA7 (AT2G35650), AtCSLA9 (AT5G03760), AtCSLA10 (AT1G24070), AtCSLA11 (AT5G16190), AtCSLA14 (AT3G56000), and AtCSAL15 (AT4G13410); OsCSLA1 (LOC_Os02g09930), OsCSLA2 (LOC_Os10g26630), OsCSLA3 (LOC_Os06g12460), OsCSLA4 (LOC_Os03g07350), OsCSLA5 (LOC_Os03g26044), OsCSLA6 (LOC_Os02g51060), OsCSLA7 (LOC_Os07g43710), OsCSLA9 (LOC_Os06g42020), and OsCSLA11 (LOC_Os08g33740); DoCSLA1 (AIW60927.1), DoCSLA2 (AIW60928.1), DoCSLA3 (AIW60926.1), DoCSLA4 (AIW60929.1), DoCSLA5 (XP_020695000.1), DoCSLA7 (AKF34889.1), DoCSLA8 (AKF34890.1), and DoCSLA9 (XP_020673043); AkCSLA3 (ADW77641.1). AkCSLA3, from *A. konjac*, was localized in small intracellular punctae and deposited glucomannan extracellularly in *Pichia* [19, 28]. AtCSLA2, a Golgi-localized protein [40], and AtCSLA9 are responsible for the synthesis of glucomannan in stems, and AtCSLA7 synthesizes glucomannan in embryos [14]. *Dendrobium officinale* DoCSLA9 is involved in the synthesis of mannan polysaccharides in transgenic *Arabidopsis* [12]. Three BsCSLAs (Bs03G1846, Bs03G11849, and Bs04G13044) were used for subsequent functional verification. **b** SALAD analysis of BsCSLAs, AtCSLAs, OsCSLAs, DoCSLAs, and AkCSLA3.

Genes that are expressed highly or preferentially in 1P would be the most promising candidates for BSP synthesis. Glucomannan in the pseudobulb tissue of *B. striata* is mostly intracellular polysaccharide (BSP) and is partly located in the cell wall (Figs 1 and 2), while glucomannan is mainly found in the stem cell wall in *Arabidopsis* [14]. Previous work showed that *SUC*, *INV*, *SUS*, *scrK*, *manA*, *PMM*, *GMPP*, *HK*, *pgm*, *UGP2*, *GPI*, and *CSLA* are involved in the synthesis of cell wall glucomannan [15, 25, 39]. To dissect the functional differentiation of these enzyme gene families in pseudobulb tissue, we first isolated these genes for analysis of the phylogenetic relationship of the homologs between BSP-rich *B. striata* and the dicot *Arabidopsis* and monocot rice, which preferentially present glucomannan in the cell wall, and other intracellular glucomannan-rich species, such as *Dendrobium officinale*, which is a member of the orchid family, and konjac (Supplementary Data Table S3; Supplementary Data Figs S10–S20; Fig. 3). Jiang *et al*. [1] assembled the haplotype genome of *B. striata* with a genome size of 2.37 Gb in haplotype A and 2.43 Gb in haplotype B (v. 1.0), in which only 26 673 and 26 891 protein-coding genes were predicted, respectively [1]. The annotated gene number was relatively small [41]. Therefore, we reassembled the genome of *B. striata* with a genome size of 2 542 457 643 bp containing 16 pseudochromosomes and finally annotated 41 311 genes (v. 2.0, unpublished data of our team). A total of 96 glucomannan-related enzymes were identified in v. 2.0, and the number of gene family members involved in glucomannan biosynthesis was analyzed in *B. striata*, rice, and *Arabidopsis*. The results showed that most gene families were expanded in *B. striata*, especially *SUC*, *scrK*, *HK*, *manA*, *PMM*, *GMPP*, and *pgm* (Supplementary Data Table S3).

In the neighbor-joining phylogenetic tree, the SUC family of *B. striata* is more closely related to monocot rice and forms different clusters with the dicot *Arabidopsis*, and the topology was supported by the SALAD analysis (Supplementary Data Fig. S10). Meanwhile, the CSLA family is divided into three branches (clusters I, II, and III). Two of the nine CSLA members, Bs04G13044 and Bs05G15273, are the closest homologs to AtCSLA2 and AtCSLA9, which are responsible for the synthesis of all detectable glucomannan in *Arabidopsis* stems [14]. Together with two rice homologs (OsCSLA1 and OsCSLA9) and one *D. officinale* protein (DoCSLA9, the name of DoCSLA6 in He *et al*. [12], which can contribute to the mannose content of water-soluble polysaccharides in transgenic *Arabidopsis*), they form a sub-branch belonging to cluster I, which covers all *Arabidopsis* CSLAs. Five of the nine rice CSLAs are in cluster III, which only contains two homologs of *B. striata* and *D. officinale*, respectively, while many *B. striata* and *D. officinale* CSLAs (five of nine and five of eight members, respectively) were concentrated in cluster II, which also contained two rice CSLAs and konjac AkCSLA3 (in which another enzyme produces glucomannan [28]), indicating these CSLAs may be a promising candidate responsible for the biosynthesis of water-soluble polysaccharides (Fig. 3). The remaining enzyme gene families in *B. striata*, rice, and *Arabidopsis* homologs were not significantly distributed in different branches (Supplementary Data Figs S11–S20), suggesting that the synthesis of BSP and cell wall glucomannan may share similar metabolic flow and enzymes except CSLA.

**Figure 4:**
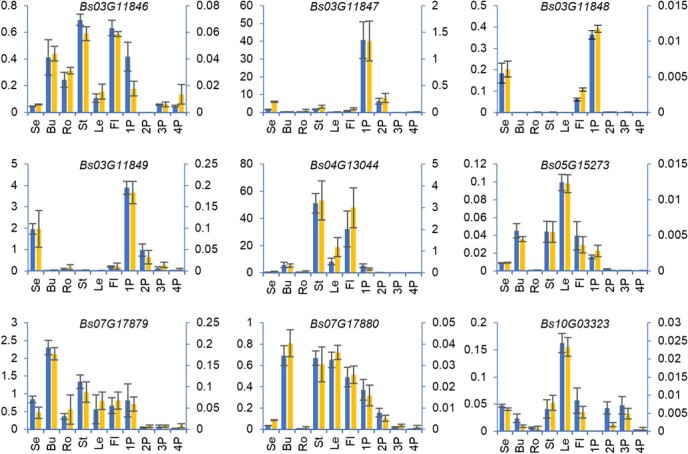
Expression profiles of nine *CSLA*s in *B. striata*. Expression levels of CSLA in 10 tissues of *B. striata* are shown relative to *UPL1* (left) and *ACT1* (right). Housekeeping genes *UPL1* and *ACT1* were used as reference genes for normalization. Values represent mean ± standard deviation (*n* = 3). Se, seedlings; Bu, buds; Ro, roots; St, stems; Le, leaves; Fl, flowers.

**Figure 5:**
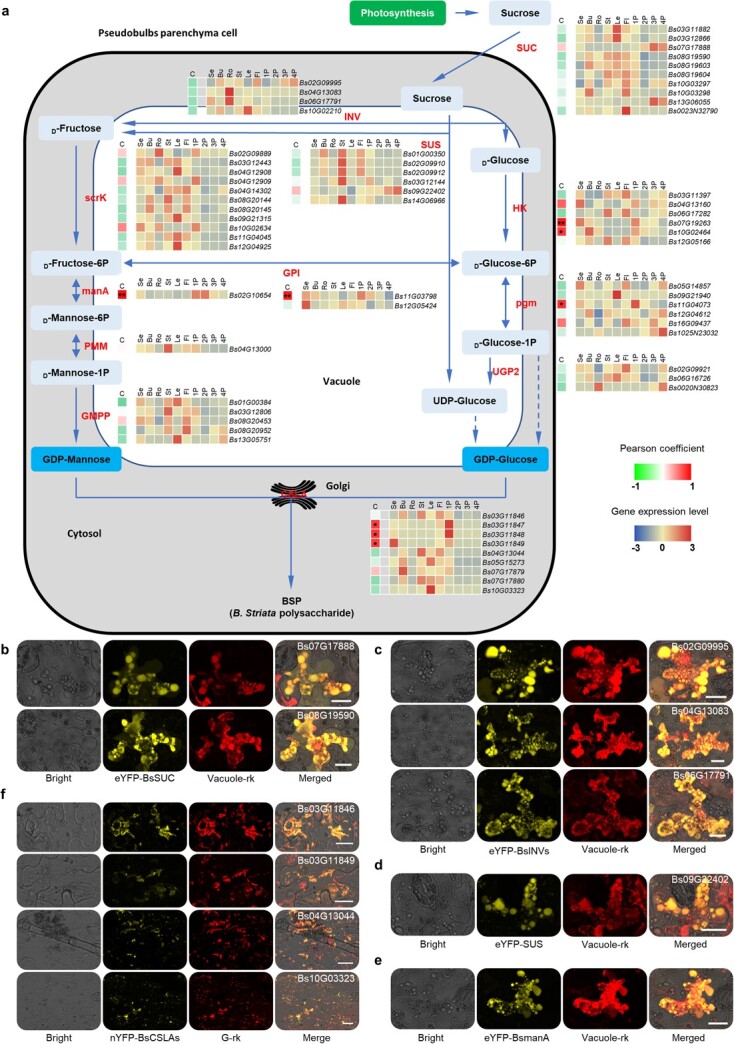
Characterization of BSP-related genes. **a** Proposed pathways for BSP biosynthesis in *B. striata*. SUC, sucrose-proton symporter; INV, invertase or β-fructofuranosidase; SUS, sucrose synthase; scrK, fructokinase; manA, mannose-6-phosphate isomerase; PMM, phosphomannomutase; GMPP, mannose-1-phosphate guanylyltransferase; HK, hexokinase; pgm, phosphoglucomutase; ugp2, UTP-glucose-1-phosphate uridylyltransferase; GPI, glucose-6-phosphate isomerase; CSLA, cellulose synthase like A. Heat map depicting the correlation (C, Pearson coefficient) between gene expression level and polysaccharide content (left) and gene expression profiles of polysaccharide (glucomannan) biosynthesis-related genes in 10 tissues of *B. striata* (right). Se, seedling; Bu, buds; Ro, roots; St, stem; Le, leaves; Fl, flowers. ^*^P < 0.05; ^**^P < 0.01. **b** Subcellular localization assay of two representative sucrose transporter (SUC) members (Bs07G17888 and Bs08G19590). The fusion construct of enhanced yellow fluorescent protein (eYFP) and test protein was driven by the cauliflower mosaic virus (CaMV)-35S promoter and was transiently co-expressed with the fusion protein of the vacuole marker (vacuole-rk) and mCherry in tobacco (*N. benthamiana*) leaf epidermal cells. **c** Subcellular localization assay of three representative invertase (INV) members (Bs06G17791, Bs04G13083, and Bs02G09995) co-expressed with vacuole-rk in tobacco leaves. **d** Subcellular localization assay of sucrose synthase (SUS) Bs09G22402. **e** Subcellular localization assay of Man-6-phosphate isomerase (manA) Bs02G10654. **f** Subcellular localization assay of four representative CSLA members (Bs03G11846, Bs03G11849, Bs04G13044, and Bs10G03323) co-expressed with the Golgi marker (G-rk) in tobacco leaves. Scale bars = 20 μm.

Following that, we constructed gene expression profiles from RNA-seq data to further screen genes expressed specifically or predominantly in the pseudobulb, where FPKM (fragments per kilobase of transcript sequence per million base pairs sequenced) >1 in any sample was the threshold to filter out some of the low-expressed genes. The results revealed that two *SUC* genes, which encode enzymes responsible for sucrose transport, *Bs03G12866* and *Bs07G17888*, are predominantly expressed in 1P, with FPKM of 28.06 and 129.79, respectively. In the *INV* and *SUS* families, which are responsible for the hydrolysis of sucrose into monosaccharides, three *SUS* genes (*Bs03G12144*, *Bs09G22402*, and *Bs14G06966*) are highly expressed in 1P, the FPKM being 239.35, 126.64, and 364.72, respectively. Three *scrK*s (*Bs02G09889*, *Bs04G12909*, and *Bs10G02634* with FPKM of 64.47, 35.67, and 33.79 in 1P, respectively), one *manA* (*Bs02G10654*, FPKM = 142.44 in 1P), one *PMM* (*Bs04G13000*, FPKM = 97.91 in 1P), and two *GMPP*s (*Bs01G00384* and *Bs03G12806* with FPKM of 42.11 and 40.60 in 1P, respectively), responsible for the synthesis of GDP-mannose, one *HK* (*Bs04G13160* with FPKM of 74.61 in 1P), three *pgm*s (*Bs05G14857*, *Bs11G04073*, and *Bs16G09437* with FPKM of 39.85, 100.12, and 32.75 in 1P, respectively), and one *UGP2* (*Bs02G09921* with FPKM of 113.01 in 1P), responsible for the synthesis of GDP-glucose, and two *GPI* genes, *Bs11G03798* and *Bs12G05424* with FPKM of 51.78 and 51.47 in 1P, respectively, responsible for the interconversion of d-Glc-6P and d-Fru-6P, are specifically or predominantly expressed in 1P. The enzyme cellulose synthase like A, encoded by *CSLA*, is responsible for the biosynthesis of the backbone of glucomannan, the key active ingredient of *B. striata*. *BsCSLA*s showed different expression profiles in different tissues and pseudobulbs of different developmental stages. *Bs10G03323* and *Bs07G17879* were preferentially expressed in leaves and buds, respectively; *Bs03G11847* and *Bs03G11848* were preferentially expressed in 1P; and *Bs03G11849* was preferentially expressed in buds and 1P (Fig. 5). However, the highest expressed *CSLA*s in 1P were *Bs03G11846* and *Bs07G17880*, with FPKM values of 22.44 and 16.37, respectively. The expression profiles of *CSLA* were also confirmed by qRT–PCR using the housekeeping genes *UPL1* and *ACT1* as the reference genes (Fig. 4).

In addition, we analyzed the correlation between polysaccharide content and the expression levels of glucomannan synthesis-related genes in roots, stems, leaves, and 1P, 2P, 3P, and 4P. The results showed that a total of eight glucomannan synthesis-related genes had high Pearson correlation coefficients with the polysaccharide content, including one *manA* (*Bs02G10654*, 0.944), two *HK*s (*Bs07G19263* and *Bs10G02464*; 0.918 and 0.787, respectively), one *pgm* (*Bs11G04073*; 0.8112), one *GPI* (*Bs11G03798*; 0.891), and three *CSLA*s (*Bs03G11847*, *Bs03G11848*, and *Bs03G11849*; 0.767, 0.785, and 0.754, respectively), suggesting that these genes might be promising candidates for BSP synthesis-related genes (Supplementary Data Table S5; Fig. 5).

Finally, the location of BSP biosynthesis was revealed by testing the subcellular localization of several major enzymes involved in BSP biosynthesis using a transient expression system in the lower epidermis of tobacco. For the sucrose transporter (SUT) enzymes, we chose Bs07G17888 and Bs08G19590 to test their subcellular localization because the former has a high expression level in 1P and is most likely a candidate involved in the BSP-synthesis pathway, whereas the latter can be used as a control to probe sucrose transport involved in an uncertain pathway. The enhanced yellow fluorescent protein (eYFP; N-terminal) fused protein of two SUCs was localized in the vacuole, implying that the sucrose transporter transported the sucrose from the source to the sink vacuole (Fig. 5c). The fusion protein signal of two gene families responsible for sucrose hydrolysis, eYFP-BsSUS (Bs09G22402) and eYFP-BsINVs (Bs02G09995, Bs04G13083, and Bs06G17791), appeared where the signal of the vacuole marker appeared (Fig. 5d and e). We performed subcellular localization analysis with manA, which is responsible for the interconversion of d-Fru-6P and d-Man-6P, as a representative gene in the monosaccharide conversion pathway, and the results showed that eYFP-Bs02G10654 was localized to the vacuole (Fig. 5f). CSLA elongates cell wall (gluco)mannans alone or together with MSR in the Golgi apparatus [28]. Prediction analyses using the online software Plant-mPLoc showed that all BsCSLAs were localized on the Golgi, and eYFP-BsCSLAs were also localized to the Golgi when transiently expressed in tobacco leaves (Fig. 5g; Supplementary Data Fig. S21). These results indicated that the glucomannan metabolic pathway, probably starting with the transport of sucrose into sink cells, undergoes a multistep enzymatic reaction on the vacuole to form the active forms of monosaccharides (GDP-Man and GDP-Glc), followed by polymerization to polysaccharides in the Golgi, and determines its final site of deposition, whether as an intracellular BSP or as a cell wall component (Fig. 5a).

In conclusion, using combined phylogenetic analysis, expression profiles, association analysis, and protein subcellular localization, we mapped the sucrose-to-BSP biosynthesis pathway and identified related gene families in *B. striata* (Fig. 5a), providing many candidate genes for further functional studies. All the results provide valuable information for the promotion of further genetic improvement of important traits in *B. striata* molecular breeding programs. Each analysis supported the importance of CSLAs, which may be the key enzymes in BSP synthesis, and subsequent experiments were performed about CSLA families.

### *Bs03G11846* and *Bs03G11849* are involved in the biosynthesis of *B. striata* polysaccharide

**Figure 6:**
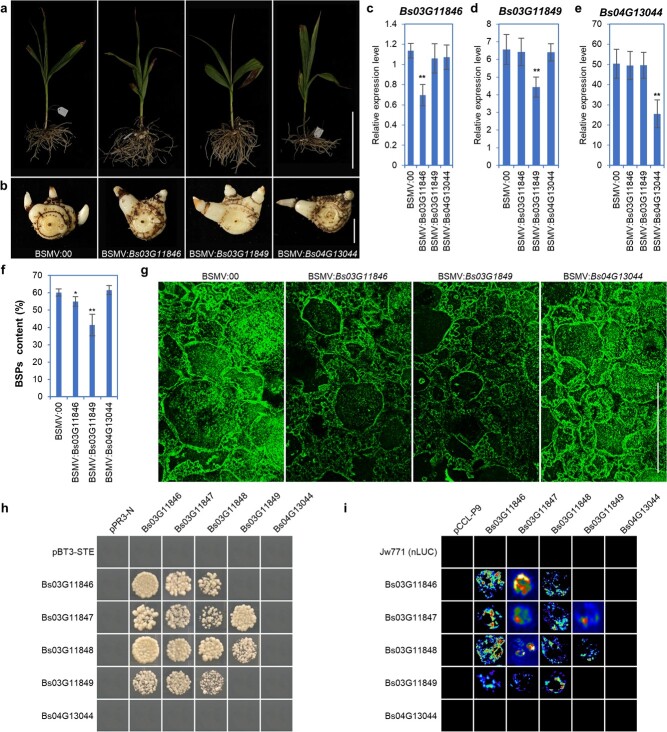
Functional identification of *Bs03G11846*, *Bs03G11849*, and *Bs04G13044* in the synthesis of BSPs in pseudobulbs of *B. striata*. **a**, **b** Plants and pseudobulbs of the empty vectors (BSMV:00, control), *Bs03G11846* (BSMV:*Bs03G11846*), *Bs03G11849* (BSMV:*Bs03G11849*), and *Bs04G13044* (BSMV:*Bs04G13044*) of VIGS transgenic lines. **c**–**e** RT–qPCR analysis of *Bs03G11846* (**c**), *Bs03G11849* (**d**), and *Bs04G13044* (**e**) transcript levels in pseudobulbs of each transgenic line. The *B. striata actin* gene (*BsACT1*) was used as the internal control for normalization. **f** BSP content of pseudobulbs of VIGS transgenic lines. **g** Immunostaining of glucomannan using monoclonal antibody LM21. **h** Luciferase complementation assay of BsCSLA proteins. *Bs03G11846*, *Bs03G11847*, *Bs03G11848*, *Bs03G11849*, and *Bs04G13044* were cloned into JW771 and pCCL vectors, corresponding to the formation of BsCSLA-nLUC and BsCSLA-cLUC, respectively. The combination with the empty vector served as a negative control. **i** Interactions among the BsCSLA proteins were analyzed by the DUALmembrane pairwise system yeast two-hybrid assay. *Bs03G11846*, *Bs03G11847*, *Bs03G11848*, *Bs03G11849*, and *Bs04G13044* were cloned into pBT3-STE and pPR3-N vectors. Transformants co-transformed with two different vectors were assayed for growth on quadruple dropout + 5 mM 3-AT nutritional selection medium. The combination with the empty vector served as a negative control. Values are the mean ± standard deviation of three biological replicates. Student’s *t*-tests demonstrated significant differences (^*^P < 0.05; ^**^P < 0.01) between the control and the *Bs03G11846*, *Bs03G11849*, and *Bs04G13044* VIGS transgenic lines. Scale bars = 20 cm in **a**, 2 cm in **b**, and 250 μm in **g**.

To further investigate which CSLA(s) are involved in the biosynthesis of BSP, we selected three *BsCSLA*s (*Bs03G1846*, *Bs03G11849*, and *Bs04G13044*) on each branch (cluster III, cluster II, and cluster I, respectively) of the phylogenetic tree as representative genes to analyze whether it is possible to influence the glucomannan level of pseudobulbs in *B. striata* by virus-induced gene silencing (VIGS). *Bs03G11846* is the highest expressed CSLA in 1P, *Bs03G11849* is the highest expressed gene of three 1P predominantly expressed genes, and *Bs04G13044* is predominantly expressed in the stem, and also has the closest relationship with *Arabidopsis* stem glucomannan-related homologs AtCSLA2 and AtCSLA9 (Figs 3, 4 and 5a). There was no obvious change in plant height and pseudobulb size between different genotypes (Fig. 6a and b). Quantitative PCR using cDNA reverse-transcribed from the RNA extracted from pseudobulbs as a template showed that the VIGS vectors were able to reduce the transcript levels of the target genes specifically and significantly without affecting the transcript levels of the homologous genes. When we examined the transcript levels of *Bs03G11846*, *Bs03G11849*, and *Bs04G13044* in each genotype, plants transgenic for the non-target genes could be used as a control, in addition to BSMV:00 (Fig. 6c and d). Both the aqueous alcoholic precipitation method for extraction and phenol-sulfate determination assay and LM21-recognized IHC assays confirmed that the content of BSPs in pseudobulbs was significantly reduced in the *Bs03G11846* and *Bs03G11846* VIGS lines (Fig. 6e and f), implying that *Bs03G11846* and *Bs03G1184* are the key genes for the synthesis of BSPs. Since both *Bs03G11846* and *Bs03G11849* play important roles in BSP synthesis, we employed DUALmembrane pairwise system yeast two-hybrid (Y2H) and luciferase complementation assays to investigate whether these two enzymes interact with each other and form homo- or heterodimers with other CSLA proteins. Two 1P-predominantly expressed CSLA members, *Bs03G11847* and *Bs03G11848*, were added to the protein–protein interaction assays. As shown in Fig. 6h, Bs03G11849 can form heterodimers with Bs03G1846, Bs03G11847, and Bs03G11849 but cannot form homodimers with itself. Bs03G11836, Bs03G11847, and Bs03G11848 can form both homodimers and heterodimers with each other among the four CSLA proteins, including Bs03G11849. The interaction of Bs11846 with Bs03G11849 is also affected by conformation, Bs03G11849-nLUC can interact with Bs03G11846-cLUC, and vice versa. Bs04G13044 was excluded from the ability to interact, as with the negative control. Luciferase complementation assays in tobacco cells confirmed the Y2H results (Fig. 6i). These data indicate that Bs03G11846 and Bs03G11849 may form both homo- and heterodimers to perform their function in BSP biosynthesis in pseudobulbs.

## Discussion

Polysaccharide is the key bioactive ingredient of *B. striata*, and nearly all the results confirmed that BSP (glucomannan) was polymerized by mannose and glucose, with molecular ratios ranging from 8.09:1 to 1.6:1 [9, 10]. In this study, the ratio of monosaccharide residues of crude polysaccharide in pseudobulb tissues of different growth years was close to 3:2, which was consistent with Chen *et al*. [10] and Wu *et al*. [42] (Table 1). Plant polysaccharides can be divided into intracellular, cell wall, and extracellular polysaccharides according to their location in plant cells. Cell wall polysaccharides located in the apoplast, including cellulose, hemicellulose, and pectin, act as structural polymers that function to support plant growth and transport water. Cellulose is synthesized on the plasma membrane mediated by the cellulose synthase complex; other cell wall polysaccharides are mainly synthesized in mobile Golgi stacks by glycosyltransferases and secreted to the cell wall by vesicle trafficking proteins [43, 44]. Extracellular polysaccharides, such as frankincense resin and peach gums, are mainly produced from its internal tissues when subjected to external invasion, and the components are galactan, glucuronic acid, mannan, xylan, and other polysaccharides [45, 46]. Peach gums may come from the degradation of parenchyma cells around the periderm and vascular cambium, while the detailed formation mechanism remains unclear [47]. Intracellular polysaccharides are considered storage polysaccharides in solution, or highly watery states exist in vacuoles (except starch), mainly mannans, fructans, and glucans, such as konjac and *B. striata* polysaccharides, both of which are glucomannans [11] **(**Table 1; Fig. 2). Agave stems present a rich content of fructans [48]. The signal of glucomannan in konjac corm tissues was evenly distributed throughout the idioblasts, initiating at the periphery and proceeding inward toward the center of the corm [11]. The quick freezing–vacuum freeze-drying of Japanese yam tubers revealed that soluble polysaccharides were the decomposition product of starch granules [49]. Our results showed that the BSPs in pseudobulbs are in the cytoplasm outside the vacuole of idioblasts of the parenchyma (Fig. 3). The 1-year-old pseudobulb has the highest content of BSP and the lowest starch granule density during the pseudobulb’s developmental stages (Supplementary Data Fig. S4).

The RNA-seq data of different tissues and their different developmental stages play an important role in revealing the spatiotemporal expression profile of genes [50]. Expression profiling has been widely used to identify candidate genes expressed in particular cell types [51, 52]. Functionally related genes usually have similar expression patterns, such as genes regulated by a common transcription factor, genes whose products constitute the same protein complex, or genes involved in the same biological process. The hierarchical cluster analysis of DEGs showed that there are many transcripts specifically or preferentially expressed in pseudobulbs (Supplementary Data Fig. S6). KEGG analysis of DEGs between 1-year-old pseudobulb and other, non-pseudobulb tissues are enriched in the map00051 and/or map00500 pathway (Supplementary Data Table S2). Furthermore, we also found that DEGs are enriched in the pathway of stilbenoid, diarylheptanoid and gingerol biosynthesis (Supplementary Data Table S2), and these compounds have been reported to have a variety of biological activities [53, 54]. These results laid the foundation for genetic research in *B. striata*, especially the molecular mechanism of medicinal active ingredients.

RNA-seq data showed that the fructose and mannose metabolism (map00051) and the starch and sucrose metabolism (map00500) KEGG pathways were enriched in 1-year-old pseudobulbs, and biochemical analyses clarified that the BSP is glucomannan. Thus, a total of 12 enzymes involved in the glucomannan biosynthesis pathway were subjected to gene family analysis (Supplementary Data Table S3), including gene expansion, phylogenetic analysis, expression profiles, and correlation analysis with BSP content. Phylogenetic tree analysis showed that the *SUC* and *CSLA* genes of *B. striata* may be functionally diverged from *Arabidopsis* and rice (Fig. 3; Supplementary Data Fig. S10). Gene expression profiling and association analysis showed that one *manA*, two *HK*s, one *pgm*, one *GPI*, and three *CSLA*s were predominantly expressed in 1-year-old pseudobulbs and had a high correlation with BSP content (Fig. 5a). The manA and GPI enzymes catalyze the interconversion of Man-6P and Fru-6P, and Fru-6P and Glc-6P, respectively, indicating that there are abundant mannose transformations in the pseudobulbs. CSLA glycosyltransferases synthesize the glucomannan backbone in *Arabidopsis* [14] and have been reported in *D. officinale* [12] and *A. konjac* [19]. Thus, all the results suggest that CSLA may be critical for BSP biosynthesis.

Although the water-soluble polysaccharide content in pseudobulbs of *B. striata* of different geographic populations ranges from 8.34 to 60.00% with an average of 45.12% [8], which is higher than 36.68 ± 0.83% in the stem of *D. officinale* [12], *B. striata* has no advantage in the number of CSLA genes. There are nine, nine and eight CSLA genes in *Arabidopsis*, rice, and *D. officinale*, respectively [12, 14]. Different subcellularly localized enzymes affect different enzymatic reactions [55, 56]. Four of five OsSUTs were plasma membrane-localized enzymes, and this set of SUT genes may compensate for their functions, while vacuole membrane protein (OsSUT2) mediates sucrose flux from the vacuole into the cytosol [56]. CSLA-encoded mannan synthases catalyze the biosynthesis of glucomannan backbone, a hemicellulose component of cell wall, in Golgi [25, 27, 40]. Zhang *et al*. speculated that GM and GGM, the major medicinal polysaccharides in *Dendrobium catenatum*, were also synthesized in the Golgi [57]. The subcellular localization analysis of all BsCSLAs showed that they were also localized in the Golgi (Fig. 5f; Supplementary Data Fig. S21). Based on the negative results of the above analysis, we formulated a hypothesis that the functional differentiation of CSLA may be the key to the high polysaccharide content in *B. striata*. We confirmed this hypothesis by phylogenetic analysis, gene expression profile analysis, and functional validation. In the phylogenetic tree, CSLAs of *B. striata* and another glucomannan-rich orchid, *Dendrobium*, have distinctive sub-branches (Fig. 3). Two *BsCSLA*s, *Bs04G13044* and *Bs05G15273*, homologous to *AtCSLA2* and *AtCSLA9*, were preferentially expressed in stems and leaves, respectively, and four *BsCSLA*s, *Bs03G11846*, *Bs07G17879*, *Bs07G17880*, and *Bs10G03323*, in cluster II and cluster III were also non-pseudobulb-expressed; these genes may be involved in the biosynthesis of glucomannan in the cell wall of these tissues (Fig. 3) [12, 14]. Three genes, *Bs0311847*, *Bs03G11848*, and *Bs03G11849*, were preferentially expressed in 1-year-old pseudobulbs, and may be the candidate genes responsible for BSPs. When we used VIGS technology to knock down the expression level of *Bs03G11846* or *Bs03G11849*, the BSP content in pseudobulbs was decreased, whereas the expression level of *Bs04G13044* did not affect the content of BSPs (Fig. 6). Meanwhile, CSLAs involved in the biosynthesis of BSPs cannot complement the deficiency phenotype of cell wall component glucomannan in *Arabidopsis* stems of *csla2csla3csla9* triple mutant (Supplementary Data Fig. S26). In addition, the four CSLA genes highly expressed in non-pseudobulb tissues, *Bs05G15273*, *Bs07G17879*, *Bs07G17880*, and *Bs10G03323*, can complement the glucomannan-deficient phenotype in the triple mutant without affecting plant growth (Supplementary Data Figs S22–S26).

Glycosyltransferases mostly form complexes to exert their functions [58, 59]. Both CSLD2 and CSLD3 of the CSL family have the ability to catalyze mannan synthesis *in vitro*, and the catalytic ability was substantially increased after co-expression, suggesting that the two enzymes may form a complex to exert synergistic effects [60]. In the CESA subfamily of the GT2 superfamily, three different enzyme subunits interact (as a necessary condition) to form a large complex that is involved in the synthesis of cellulose deposited at different sites [61–63]. In *Arabidopsis*, cellulose catalyzed by the cellulose synthase complex consisting of AtCESA4, AtCESA7, and AtCESA8 is distributed in the secondary cell wall, whereas cellulose catalyzed by the cellulose synthase complex formed by AtCSEA1, AtCESA3, and AtCESA6 is distributed in the primary cell wall [62, 64], and evidence in species such as rice, poplar, and cotton also supports the complex model, but with some variations in subunit ratios and numbers [65]. Cotton mature fibers with >90% cellulose may benefit from having a supercomplex consisting of a larger number of enzyme subunits [66]. In our study, enzymes encoded by the CSLA genes were also able to form homo- and heterodimers involved in the biosynthesis of BSPs (Fig. 6), which may be important evidence for revealing the high water-soluble polysaccharide content in pseudobulbs.

In plants, many critical enzymes in the metabolic pathway are physically clustered in the genome. Gene clustering is reported in various types of plants and involved in many processes, such as plant development, genome architecture, and specialized metabolism [67–69]. In opium poppy, a particularly complex gene cluster contains many critical enzymes in the metabolic pathway that generates the alkaloid drugs noscapine and morphinan [68]. In rice, cytochrome P450 monooxygenase, CYP76M7, is clustered into two sets and uniquely multifunctional, with the corresponding genes being subject to distinct transcriptional regulation [67]. Coordinated regulation of gene expression is considered to be part of the selective pressure resulting in the retention of a multigenic trait as a single locus to facilitate the synthesis of the final product [69]. We found that some members of the same enzyme were clustered on the chromosome, and there are also multiple enzymes clustered together, such as some members of the SUC, SUS, scrk, and CSLA enzymes in the BSP synthesis pathway (Supplementary Data Fig. S28), where four CSLA genes (*Bs03G11846*, *Bs03G11847*, *Bs03G11848*, and *Bs03G11849*) were clustered within the 0.5-Mb sequence of pseudochromosome 3 (Supplementary Data Fig. S27). The latter three genes showed almost identical expression patterns, mainly predominantly expressed in the 1-year-old pseudobulbs, which is the most BSP-rich tissue of *B. striata*, and thus had a significant correlation with the BSP content (Fig. 5a). These genes encode enzymes that had similar protein structural domains (Fig. 3b) and were on the same evolutionary sub-branch (Fig. 3), which ultimately confirmed that silencing of these genes could affect BSP synthesis (Fig. 6). *Bs03G11846* showed similar expression characteristics to the five genes located on other chromosomes that were dominantly expressed in non-pseudobulb tissues. Two genes, *Bs07G17879* and *Bs07G17880*, were also found to be tandem genes on pseudochromosome 7 with similar expression patterns (Fig. 4), but the protein structural domains were highly differentiated and located in different evolutionary sub-branches (Fig. 3), also suggesting functional selection and differentiation of the CSLA gene cluster under evolutionary pressure.

## Materials and methods

### Plant material

All the materials of *B. striata* used in this study were derived from the HBYL population planted in the resource nursery of the Wuhan Botanical Garden of the Chinese Academy of Sciences. Selfed seeds from a 5-year-old plant of the HBYL population were successively surface-sterilized with 75% ethanol and 10% benzalkonium bromide, and washed with ddH_2_O three to five times. The sterilized seeds were sown on Murashige and Skoog (MS) solid plates with 30 g/l sucrose, 2 mg/l 6-BA, and 1 mg/l NAA at 25 ± 1°C with a 16 h light/8 h dark photoperiod. The 5-month-old seedlings contained pseudobulbs, roots, stems, and leaves, which were sampled for RNA extraction. In March, buds, roots, stems, and flowers were sampled from the 5-year-old plant. In June, 1-, 2-, 3-, and 4-year-old pseudobulbs were sampled from the same plant (Supplementary Data Fig. S1). Part of the samples was used for paraffin embedding, part for polysaccharide content determination, and the rest was immediately frozen with liquid nitrogen and then stored at −80°C for RNA extraction.

*Arabidopsis thaliana* (ecotype Columbia) seeds were surface-sterilized with 75% ethanol containing 0.1% Triton X-100 for 1 min twice, washed with sterile water three times, and placed on MS plates with 30 g/l sucrose maintained at 4°C for 2 days. Then, the plates were transferred to a habitat of 16 h light/8 h dark and 22 ± 1°C for ~7 days for germination. The seedlings were transplanted into soil for growth under the same conditions as for germination. Homozygotes of the *csla2/3/9* triple mutant were placed on MS plates, and positive seeds of *BsCSLA* overexpression transgenic lines in the wild-type and *csla2/3/9* mutant background were placed on MS plates with 50 mg/l kanamycin or 25 mg/l hygromycin.

Tobacco (*N. benthamiana*) seeds were germinated in vermiculite, and then the dominant growth seedlings were transplanted into soil at 22 ± 1°C with 16 h light/8 h dark until they grew to eight or nine leaves for transient transformation.

### Polysaccharide content and monosaccharide composition analysis

Roots, stems, and leaves of *B. striata* were dried in a 40°C oven, while the sampled pseudobulbs were sliced, steamed in a water bath, and dried in the shade according to the Chinese Pharmacopeia (edition 2020). Approximately 1 g was crushed into a fine powder sample (a) and was extracted twice with 20 ml dH_2_O at 100°C for 1 h. After adding a four times volume of absolute ethanol to the mixed extract, the resulting precipitate was dried to obtain crude BSP and weighed (b). The water-soluble polysaccharide content (%) was calculated by the formula c = b/a × 100%. Analysis of polysaccharide content using the phenol–sulfuric acid method with glucose as the standard was as described in our previous study with minor modification [8].

The monosaccharide components in crude polysaccharide were quantitatively analyzed by GC using a two-step silylation derivatization procedure, and inositol was added as an internal standard. Approximately 10 mg of crude polysaccharides was added with 50 μl 5 mg/ml inositol as an external standard, and then the polysaccharides was hydrolyzed into monosaccharides at 121°C for 2 h with 1.5 ml 2 M trifluoroacetic acid (TFA). Two hundred microliters of supernatant was dried at 40°C, 100 μl 20 mg/ml anhydrous pyridine-dissolved methoxyamine hydrochloride (CAS number 593-56-6, Sigma–Aldrich) was added and reacted at 37°C for 2 h, then 100 μl silanization reagent (BSTFA with 1% TMCS, CAS number 25561-30-2, Sigma–Aldrich) was added and reacted at 37°C for 30 min. Subsequently, the derivatized monosaccharide was determined using a comprehensive 2D GC mass spectrometer (GC-2010Plus AF/GCMS-QP2020, Shimadzu, Japan). The compounds of each peak were determined according to the retention time of the standard monosaccharides and the mass spectrometry result, and the content of each monosaccharide was calculated from the ratio of peak area to internal standard inositol peak area. As references we used the standard monosaccharides xylose, arabinose, rhamnose, mannose, glucose, and galactose, which were derived in the same way as BSP. The temperature of the column started at 160°C and was held for 1 min, then increased to 172°C at a rate of 10°C/min, increased to 208°C at a rate of 5°C/min, decreased to 200°C in 10 s, held for 2 min, and decreased to 160°C and held for 2 min.

### Paraffin-embedded sectioning and imaging of *B. striata* polysaccharides

The roots, stems, leaves, and 1-, 2-, 3-, and 4-year-old pseudobulbs of *B. striata* and the 1-cm basal part of the main inflorescence stems of *A. thaliana* were cut into small pieces with a length <0.5 cm and immediately fixed in a 2-ml formalin–acetic acid–alcohol (FAA) solution (3.7% formaldehyde, 5% glacial acetic acid, and 50% ethanol in dH_2_O) for 2 days. Then, samples were dehydrated in ethanol (75% for 4 h, 85% for 2 h, 90% for 2 h, 95% for 1 h, and 100% for 30 min twice), made transparent with xylene for 10 min twice, and embedded in paraffin. The paraffin-embedded samples were sliced into 4-μm-thick sections using an RM2016 microtome (Leica, Germany), and treated with xylene, ethanol, and dH_2_O in turn.

For β-mannanase hydrolysis, β-mannanase (M9280, Solarbio, China) was dissolved in citric acid–disodium hydrogen phosphate (CA-DHP) buffer (pH 4.8). Rehydrated sections were treated with mannanase or CA-DHP buffer for 4 h at 55°C.

Fluorescent signals of polysaccharides (glucomannan) were labeled using a protocol previously used for *Arabidopsis* stem with modifications [70]. In brief, the non-specific binding sites of sections were blocked by incubation with BSA/phosphate-buffered saline (PBS) (3% BSA in 1 × PBS) for 1 h, then incubated with rat anti-heteromannan antiserum (LM21) diluted in PBS/BSA overnight at 4°C in a humidity box. The sections were washed with three changes of PBS with 5 min for each change, and incubated with a secondary antibody diluted in the region of 200-fold in BSA/PBS for 1 h at room temperature (RT). LM21 (AS18 4209, Agrisera, Sweden) was diluted 1:20 [71], while FITC-conjugated goat anti-rat IgG secondary antibody (GB22302, Servicebio, Wuhan, China) was diluted 1:200. The sections were washed three times with PBS, allowing 5 min between changes. The samples were mounted using a small drop of anti-fade reagent (G1401, Servicebio, Wuhan, China) and covered with a coverslip. FITC fluorescence was visualized under a confocal laser scanning fluorescence microscope (TCS SP8, Leica, Germany) with excitation wavelength 488 nm and emission wavelength 520–530 nm.

### Immuno-transmission electron microscopy

The middle part of the 1-year-old pseudobulb was cut into small pieces <0.2 cm in length and immediately fixed with 2 ml of immunoelectron microscopy special fixative (G1124, Servicebio, Wuhan, China) for 2 h at RT in the dark, and then transferred to 4°C for storage. The samples were dehydrated in ethanol (30% for 20 min at 4°C, 50 and 70% progressively for 20 min at −20°C, and 80, 85, 90, 95, 100, and 100% progressively for 10 min at −20°C), which was gradually replaced by LR white resin (14381-UC) at 4°C, and finally embedded in resin at 4°C and transferred to an encapsulated capsule. The resin was polymerized in a low-temperature UV polymerizer for 48 h at −20°C. The resin-embedded sample was sliced into 70-nm-thick ultrathin sections with a microtome (UC7, Leica, Germany) and Daitome Ultra 45° knives, and the sections were collected at 4°C onto formvar-coated nickel grids BZ102615Na (150 mesh, ZhongJingKeYi, Beijing, China).

The nickel grids were thoroughly rinsed in ultrapure water for hydration, washed three times with 1 × Tris-buffered saline (TBS) for 5 min each at RT, and blocked in 1% BSA/TBS for 30 min at RT. Sections were then incubated with the primary antibody LM21 at a dilution of 1:10 in 1% BSA/TBS at 4°C overnight in a humidity box and washed three times with TBS for 5 min each at RT. Sections were incubated with a 10-nm anti-rat IgG (whole molecule)–Gold antibody produced in goat (G7035, Sigma–Aldrich) at a dilution of 1:50 in a humidity box at RT for 20 min, at 37°C for 1 h, and again at RT for 30 min. They were washed five times with TBS for 5 min each and five times with ultrapure water for 5 min each. Grids were stained with 2% (w/v) uranyl acetate saturated ethanol solution in the dark for 8 min, washed three times with 70% ethanol, three times with ultrapure water, and then dried in a 37°C oven for ~10 min. Sections were viewed with a Hitachi HT7800 transmission electron microscope at an accelerating voltage of 80 kV.

### RNA extraction and reverse transcription

Total RNA from *B. striata* tissues was isolated using the TaKaRa MiniBEST Plant RNA Extraction Kit (No. 9769, TaKaRa, Japan), and total RNA from the basal third of the mature inflorescence stems of *A. thaliana* was isolated using the TRIeasy™ Total RNA Extraction Reagent (10606ES60*, Yeasen, China). Approximately 100 mg of tissue powder ground with liquid nitrogen was used to extract total RNA, and then the RNA solution was stored at −80°C. Two micrograms of total RNA was reverse-transcribed into cDNA using SMART MMLV Reverse Transcriptase (No. 639523, Clontech) according to the manufacturer’s protocol. In brief, 2 μg total RNA was adjusted to 11 μl with RNA-free H_2_O, 1 μl Oligo(15 T) was added and incubation was performed at 70°C for 3 min. After cooling on ice, 4 μl of 5 × first-strand buffer, 2 μl of Advantage UltraPure PCR Deoxynucleotide Mix (No. 639125, Clontech), and 2 μl of 10 mM DTT were added in sequence, and then the preparation was incubated at 42°C for 60 min to obtain 20 μl of cDNA solution. The cDNA solution was stored at −20°C.

### RNA-seq analysis

The total RNA from 10 samples of *B. striata* (seedlings, buds, roots, stems, leaves, flowers, and 1-, 2-, 3-, and 4-year-old pseudobulbs) were entrusted to Annoroad to construct RNA-seq libraries and sequenced on an Illumina PE150 system, each tissue having three biological replicates. FPKM was used to estimate the expression level of transcripts. RSEM software was used to analyze the gene expression level of each sample, and FPKM = 1 was used as the threshold for gene expression. Genes with parameters of |log_2_ratio| ≥ 1 and *q* (corrected *P*-value) < 0.05 were selected as significant DEGs and annotated with NCBI, UniProt, GO, and KEGG databases. Considering the pseudobulb as the medicinal tissue, eight groups of 10 samples were selected for DEG analysis, seedlings, buds, roots, stems, and leaves were compared with the 1-year-old pseudobulb, and four developmental stages of the pseudobulb were compared with each other. We used R software (version number: v3.1.1) to perform hierarchical cluster analysis on DEGs and different tissues. The logarithm of the expression level of the DEGs in each sample to base 2 were used to calculate the Euclidean distance.

### Identification of genes related to the *B. striata* polysaccharide biosynthetic pathway

The transcripts in the annotation file matched to fructose and mannose metabolism (map00051) and starch and sucrose metabolism (map00500) of the KEGG pathway were isolated as candidate genes related to the monosaccharide substrate mannose and glucose biosynthetic pathway of BSP. CSLA may be involved in the biosynthesis of the glucomannan backbone. The gene expression level in different tissues was standardized using the formula *Y* = (FPKM-mean)/STDEV, FPKM, the FPKM of tested tissue; mean and STDEV, the average and standard deviation (SD) of the FPKM of all samples. Correlation analysis was performed by calculating the Pearson correlation coefficient between polysaccharide content and gene expression level in seven tissues, including root, stem, leaf, and 1-, 2-, 3-, and 4-year-old pseudobulbs. The hot map of gene expression profiles and correlation analysis was drawn in Microsoft Excel 2019.

Multiple sequence alignment of amino acid sequences of genes of interest performed using ClustalW with default parameters was used to infer a phylogenetic tree by the neighbor-joining method with 1000 bootstraps in MEGA 11.0. Interactive SALAD analysis (http://salad.dna.affrc.go.jp/CGViewer/en/cgv_upload.html) was applied to investigate the conserved motifs of full-length amino acid sequences of BSP-related genes.

### Quantitative real-time PCR

Twenty microliters of reverse-transcription cDNA was diluted 20 times, and 2 μl of diluted cDNA was used as template for the PCR. TB Green^®^ Premix Ex Taq™ II (No. RR820A, TaKaRa) was employed for the amplification reaction, which comprised 95°C for 1 min; 40 cycles of 95°C for 5 s, 58°C for 10 s, 72°C for 30 s, and a final elongation step at 72°C for 10 min. The formula *Y* = 10^∆Ct/3.5^ × 100% was used to calculate the relative expression of genes; ∆Ct is the Ct value difference between the reference gene and the selected gene. The housekeeping genes *ubiquitin-protein ligase* (*UPL*) and *actin* (*ACT*) were selected as the internal reference genes (Supplementary Data Table S4).

### Subcellular localization analysis

The coding sequence of the gene of interest, amplified from cDNA using specific primers (Supplementary Data Table S4) that included the restriction enzyme site SpeI by PCR with the proofreading PrimeSTAR^®^ HS DNA polymerase (No. R010A, TaKaRa), was cloned into a position downstream of the eYFP gene of the pMDC83-*eYFP* vector, which was driven by the CaMV35S promoter. The recombinant vector was transformed into *Agrobacterium tumefaciens* strain GV3101, and freshly cultured *Agrobacterium* cells were resuspended in infiltration buffer [10 mM MgCl_2_, 10 mM 2-(N-morpholino)ethanesulfonic acid (MES), pH 5.2, and 0.1 mM acetosyringone] to OD600 = 0.6 and incubated at RT for ~2 h. The *Agrobacterium* suspension containing the tested genes and the subcellular organelle markers were co-infiltrated into the abaxial side of *N. benthamiana* leaves in a ratio of 2:1 using a needleless syringe. At 3 days after infiltration, the eYFP fluorescence signals in infiltrated epidermal cells were visualized under a confocal laser scanning fluorescence microscope (TCS SP8, Leica, Germany) with excitation wavelength 514 nm and emission wavelength 527–532 nm. The tonoplast, Golgi, and plasma membrane markers were fused with mCherry protein, and the red fluorescence signals were monitored at an excitation wavelength of 552 nm and an emission wavelength of 610–615 nm.

### Complementation of *cesa2cesa3cesa9* triple mutants

*Agrobacterium* cells containing the constructed pMDC83-*eYFP*-*BsCSLAs* vectors were resuspended in transformation solution (5% sucrose, 0.025% Silwet L-77) to OD600 = 0.8 and used for dipping the *csla2csla3csla9* triple mutant plant flowers. Positive first-generation (T_1_) transgenic plants were selected on MS medium (3% sucrose) with 25 mg/l hygromycin B (No. 60225ES03, Yeasen, China) and identified by PCR. T_1_ transgenic lines were used for expression level identification. Total RNA was isolated from the basal 10 cm of the inflorescence stems of 8-week-old plants, and the expression level was analyzed by qRT–PCR as described above, in which *AtACTIN2* was used as the internal reference gene. Confirmed high-expression-level T_2_ transgenic lines were used for phenotypic analysis. The basal 0.5 cm of the main inflorescence stem of mature plants was isolated, fixed in FAA solution, and paraffin-embedded, and 4-μm-thick sections were performed to image glucomannan by LM21-labeled immunohistochemistry.

### Virus-induced gene silencing

To knock down the expression of *Bs03G11846*, *Bs03G11849*, or *Bs04G13044* in *B. striata*, VIGS technology was implemented using the *Barley stripe mosaic virus* (BSMV) system (pCaBS-*α*, pCaBS-*β* and pCa-*γb*LIC) according to an *Agrobacterium* delivery method [72, 73]. We cloned a specific fragment of *Bs03G11846* (292 bp) with the 5′-AAGGAAGTTTAA-3′ sequence added at the forward end and the 5′-AACCACCACCACCGT-3′ sequence added at the reverse end (Supplementary Data Table S4), and then digested it using T4 DNA polymerase with dATP. Simultaneously, ApaI-linearized pCa-*γb*LIC was digested by T4 DNA polymerase with dTTP. The recombinant plasmid pCa-*γb*-*Bs03G11846* was obtained by incubating the *Bs03G11846* VIGS target fragment with A sticky ends with linearized pCa-γbLIC with T sticky ends at 25°C for ~30 min. pCa-*γb*:*Bs03G11849* and pCa-*γb*:*Bs04G13044* were obtained by the same method, in which *Bs03G11849*- and *Bs04G13044*-specific fragments were 294 and 303 bp, respectively.

pCaBS-*α*, pCaBS-*β*, pCa-*γb*LIC (or its derivatives pCa-*γb*:*Bs03G11846*, pCa-*γb*:*Bs03G11849*, and pCa-*γb*:*Bs04G13044*) were transformed into *A. tumefaciens* strain EHA105. *Agrobacterium* cells were resuspended in infiltration buffer [10 mM MgCl_2_, 10 mM MES pH 5.2, and 0.1 mM acetosyringone] to OD600 = 1.0 and incubated at RT for ~2 h. Equal amounts of *Agrobacterium* cells harboring pCaBS-*α*, pCaBS-*β*, and pCa-*γb*-*Bs03G11846* were mixed as the BSMV:*Bs03G11846* group, and correspondingly produced BSMV:*Bs03G11849* and BSMV:*Bs04G13044* group. The pCaBS-*α*, pCaBS-*β*, and pCa-*γb*LIC mixed group (BSMV:00) was used as the control. The waxy epidermis of the adaxial side of *B. striata* leaves was disrupted with sandpaper, and four groups of *Agrobacterium* cells were infiltrated into the leaves and infiltrated again 7 days later. Infestation was completed in April, and the *B. striata* plants were cultured in a greenhouse (25°C) for 4 months. Pseudobulbs were collected in August for subsequent experiments, including RNA isolation, paraffin-embedded sectioning, and BSP content determination.

### Luciferase complementation assay

With the restriction enzyme sites KpnI and SalI (Supplementary Data Table S4), the coding sequences of *BsCSLA*s (without the stop codon) were cloned into a position upstream of the luciferase (LUC) C-terminal (cLUC) of the pCCL vector to produce BsCSLA-cLUC, and were cloned into a position upstream of the LUC N-terminal (nLUC) of the JW771 vector to produce BsCSLA-nLUC. Both BsCSLA-cLUC and BsCSLA-nLUC were driven by the CaMV35S promoter. All vectors were transformed into *A. tumefaciens* strain GV3101 by electroporation. *Agrobacterium* cells carrying nLUC and cLUC vectors were resuspended in infiltration buffer to OD600 = 0.5, mixed at a ratio of 1:1, then infiltrated into the tobacco abaxial epidermis as described above (see Subcellular localization analysis). Three days after infiltration, the tobacco leaves were cut off and the infiltration surface was sprayed with luciferase substrate [0.3 mg/ml d-luciferin firefly, potassium salt (No. 40902ES01, Yeasen, China), 0.1% TrotonX-100] and photographed under a chemiluminescence system. pCCL and JW771 were used as negative control.

### DUALmembrane pairwise system yeast two-hybrid assay

The coding sequences of *BsCSLA*s were cloned into a position downstream of *NubG* (the isoleucine at position 3 of N-terminal ubiquitin was exchanged for a glycine) of the pPR3-N vector with the restriction enzyme sites SfiI to produce the prey plasmid. The other tested gene (without the ATG codon and the stop codon and adding two GGs at the final 3′ end) was cloned into a position between *STE2* and *Cub-LexA-VP16* of the pBT3-STE vector with the restriction enzyme site SfiI to produce the bait plasmid (Supplementary Data Table S4). The bait plasmid was co-transformed with the prey plasmid into the NMY51 yeast reporter strain by electroporation using a 2-mm-gap gene pulser/micropulser electroporation cuvette, and each transformation was plated onto SD−Trp−Leu (SD minimal medium lacking Trp and Leu) selection plate and incubated at 30°C for 4 days. Freshly cultured transformants were diluted 100-fold with 0.9% NaCl solution, and 8 μl yeast cells were spotted on SD−Trp−Leu−Ade−His (quadruple dropout) selection medium with 5 mM 3-aminotriazole (3-AT) plate to test the interactions.

## Supplementary Material

Web_Material_uhae092

## Data Availability

The data underlying this article are available in the article and in its online supplementary information. Genomic data (PRJCA022253) and transcriptomic data (PRJCA022233) are deposited at the China National Center for Bioinformation (https://www.cncb.ac.cn/), and the big data sets are also available from the corresponding author.
