# Supplementary material for: Cytochemical localization and synthesis mechanism of the glucomannan in pseudobulbs of Bletilla striata Reichb. f
Source: Hortic Res. 2024 Apr 22;11(5):uhae092. doi: 10.1093/hr/uhae092 (PMC11116825; doi:10.1093/hr/uhae092)
Supplement: Web_Material_uhae092 [file web_material_uhae092.zip › Supplementary figures.docx]

**
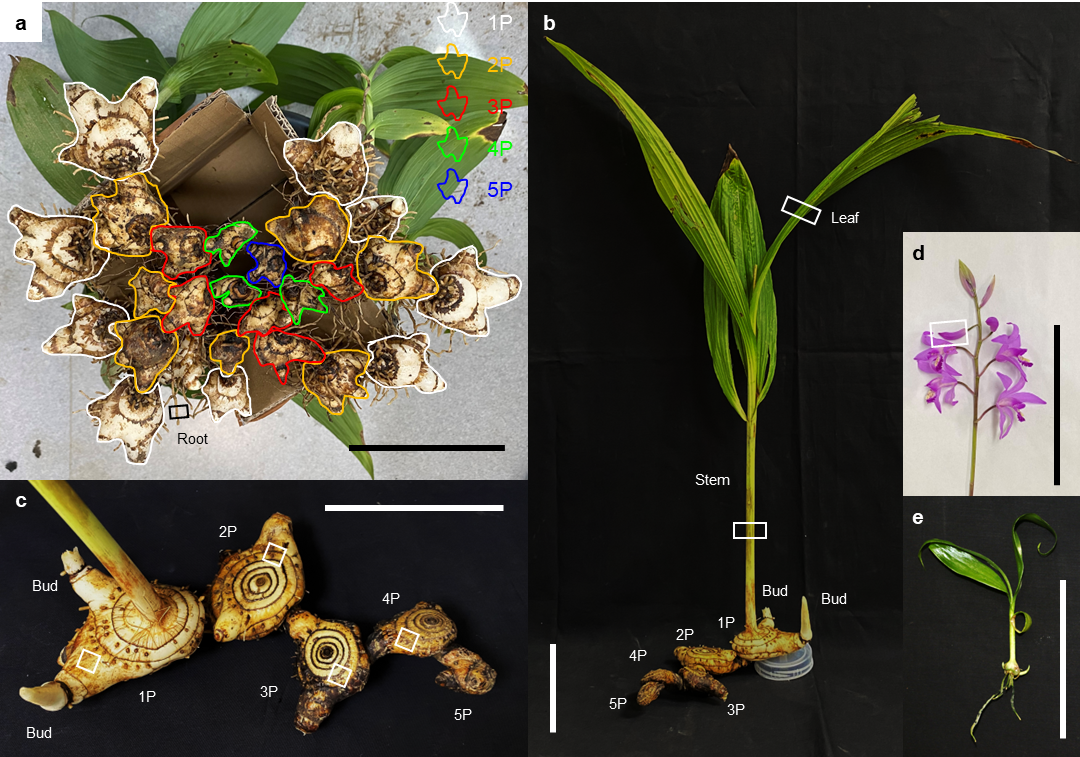
**

**Supplementary Figure S1** **Morphology of *B. striata*.** (**a**) A 5-year-old plant (inverted, roots removed) of *B. striata* from the HBYL population. (**b**) Plants that remove redundant pseudobulbs and roots, leaving one pseudobulb of each growth year. (**c**) An enlarged view of the underground part in **b**. 2-3 buds grow from the one-year-old pseudobulbs in September and develop into stems, leaves, and reproductive tissues in the next year. 1P, 2P, 3P, 4P, and 5P represent one-, two-, three-, four-, and five-year-old pseudobulbs, respectively. (**d**) Inflorescence. (**e**) Seedlings of selfed HBYL population contained roots, pseudobulbs, stems, and leaves. The box represents the site used for paraffin embedding and RNA extraction. Scale bars = 10 cm.


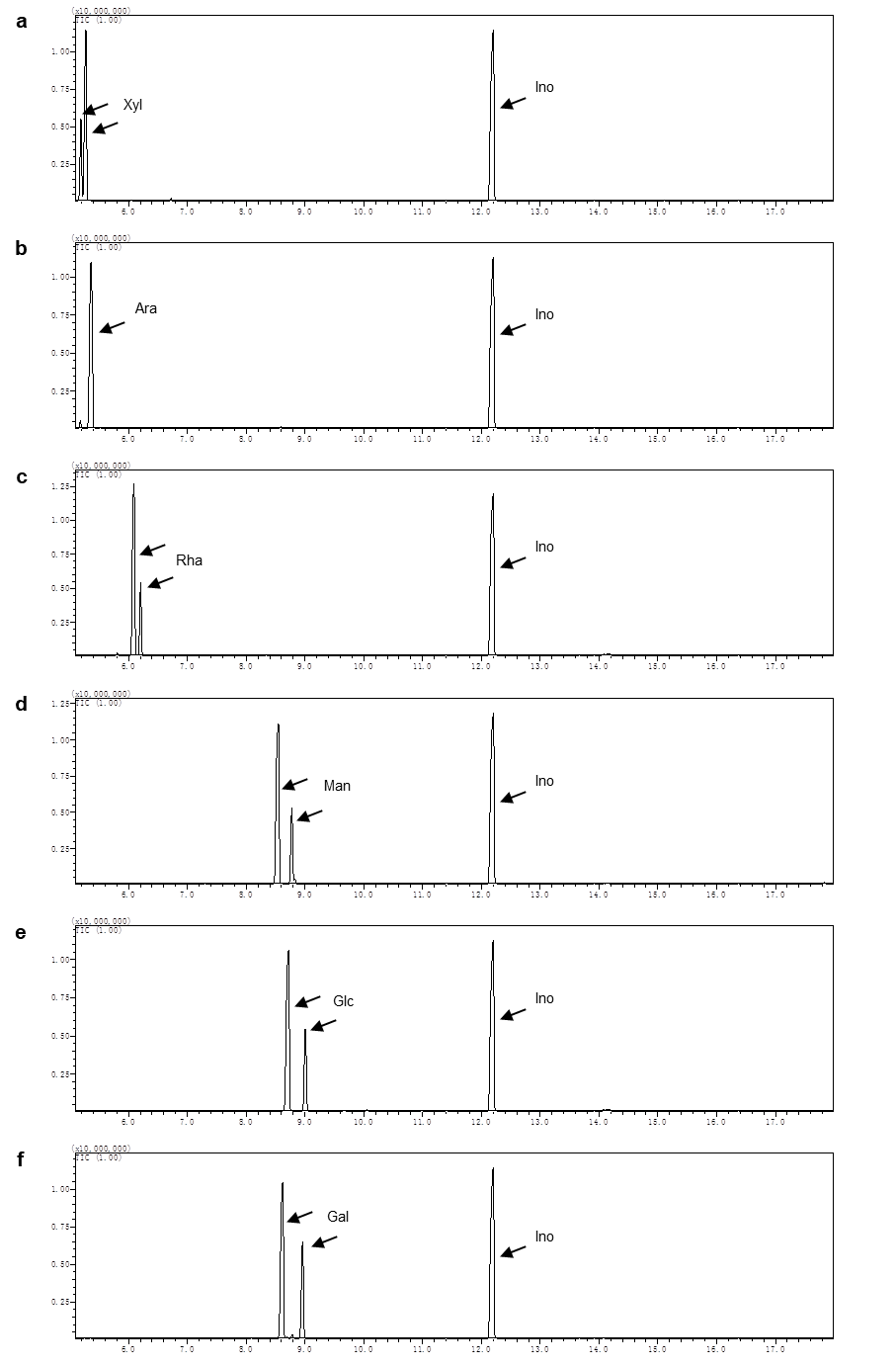


**Supplementary Figure S2** **Chromatogram of reference monosaccharide. a**, Xyl, xylose; **b**, Ara, arabinose; **c**, Rha, rhamnose; **d**, Man, mannose; **e**, Glc, glucose; **f**, Gal, galactose. Inositol (Ino) as an exogenous internal reference.


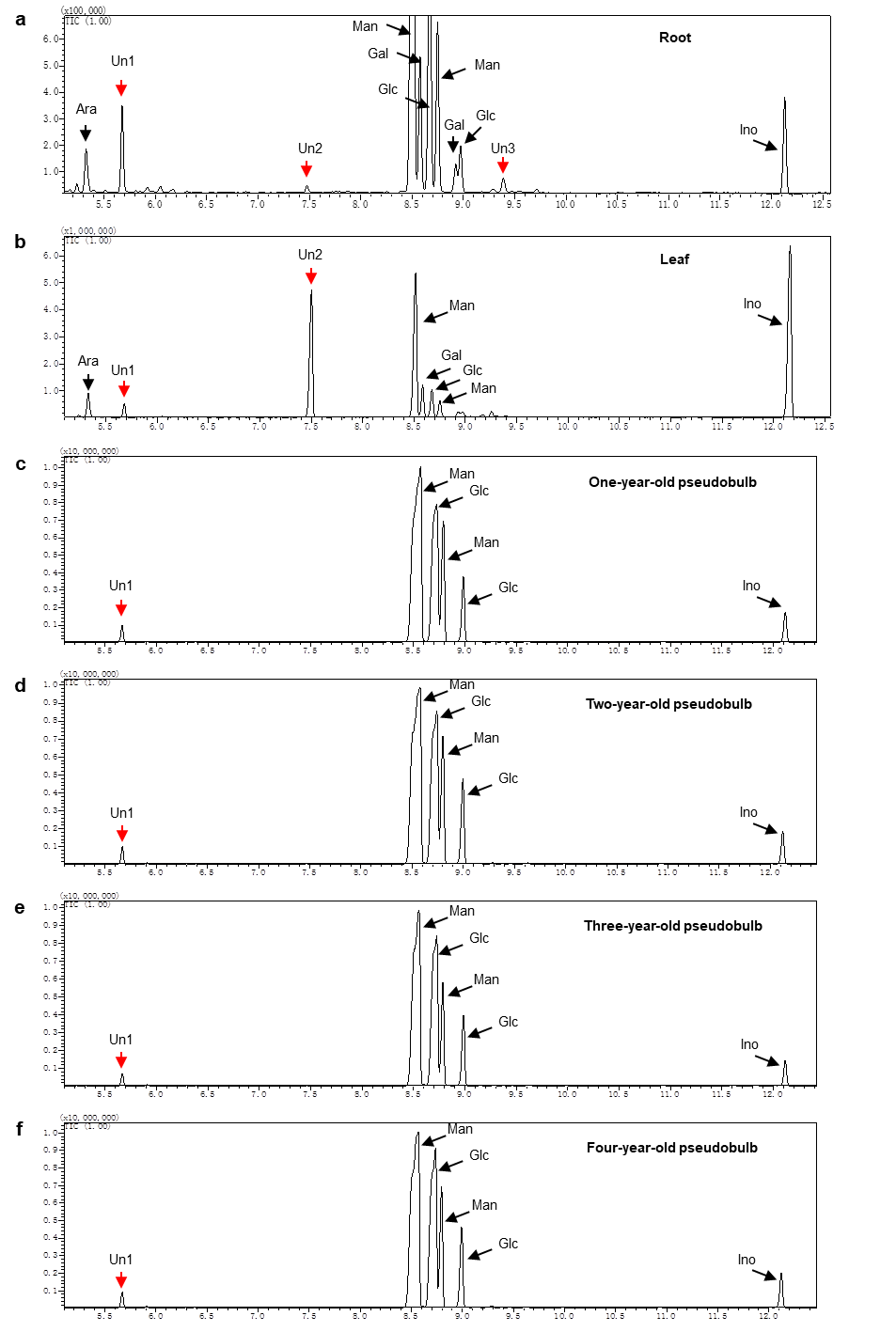


**Supplementary Figure S3** **Gas chromatograms of crude polysaccharides from roots, leaves, and pseudobulbs at different growth stages after hydrolysis by TFA and subsequent two-step silylation derivatization. a**, root; **b**, leaf; **c**, one-year-old pseudobulb; **d**, two-year-old pseudobulb; **e**, three-year-old pseudobulb; **f**, four-year-old pseudobulb. Ara, arabinose; Man, mannose; Glc, glucose; Gal, galactose; Ino, inositol. Un1, Un2 and Un3 represent unknown peaks.


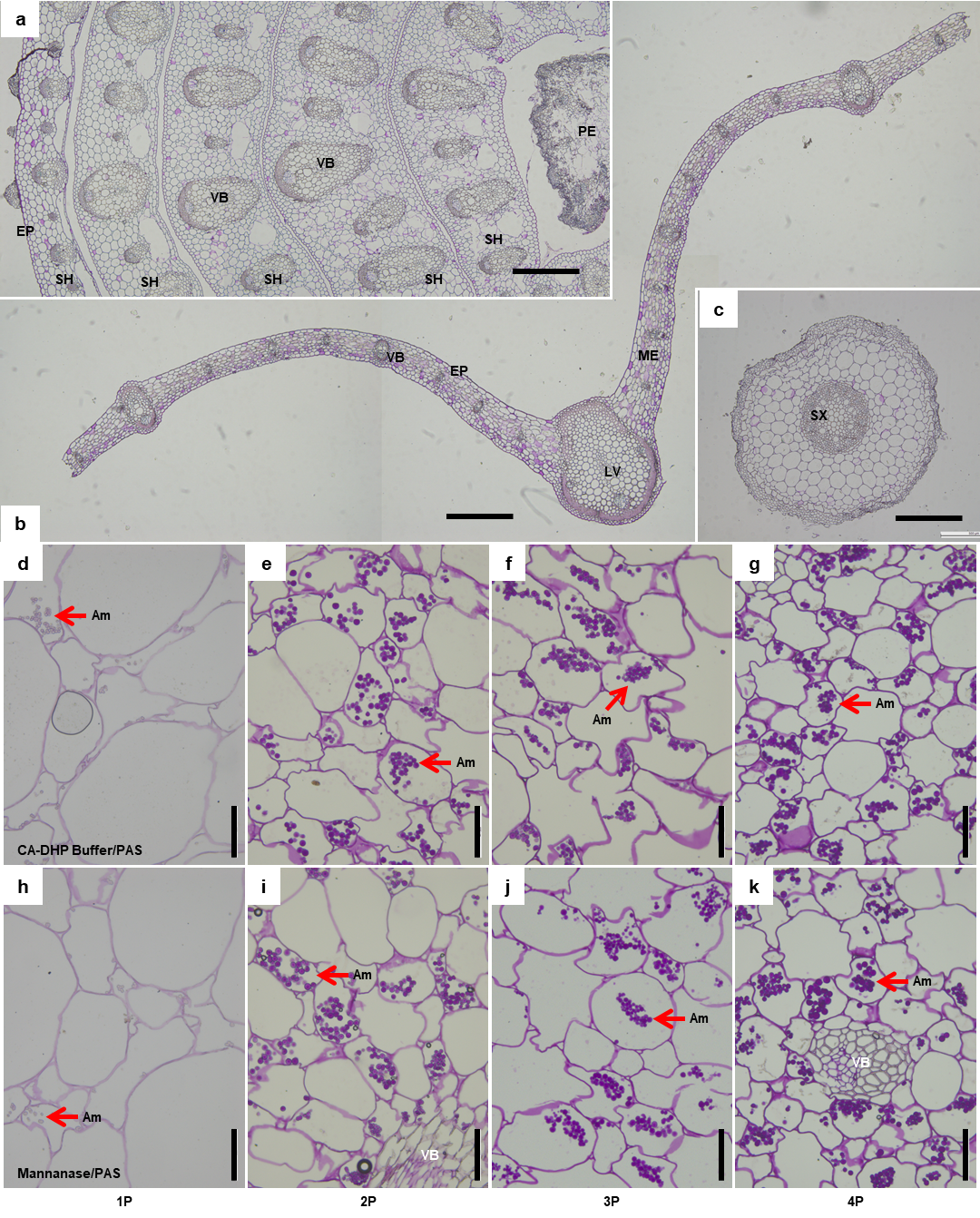


**Supplementary Figure S4** **Light micrographs of root (c), stem (a), leaf (b), and pseudobulbs (d-k) stained with the periodic acid-Schiff (PAS) reagent.** Signal intensity in pseudobulbs is not proportional to polysaccharide content (**d**-**g**) and does not disappear with the addition of mannanase (**h**-**k**). Ep, epidermis; sh, leaf sheath; pe, peduncle; vb, vascular bundle; me, mesophyll; lv, leaf vein; sx, secondary xylems. Bars = 100 μm.


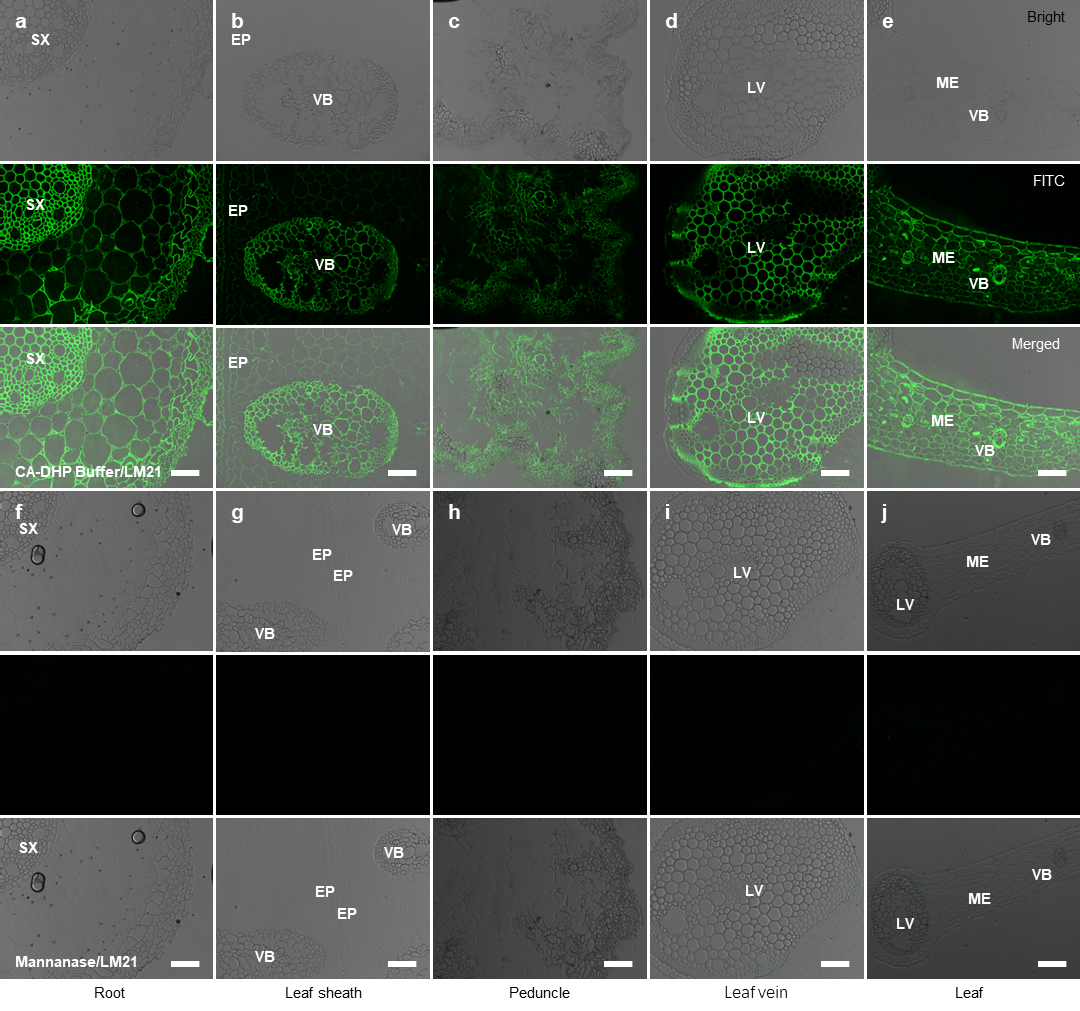


**Supplementary Figure S5** **Immunolocalization of glucomannan labeled by monoclonal antibody LM21 in root, stem, and leaf of *B. striata*. f**-**j** and **a**-**e**, pre-treated with ß-mannanase or not in immunolocalization, respectively. sx, secondary xylems; ep, epidermis vb, vascular bundle; lv, leaf vein; me, mesophyll. Bars = 100 μm.

**
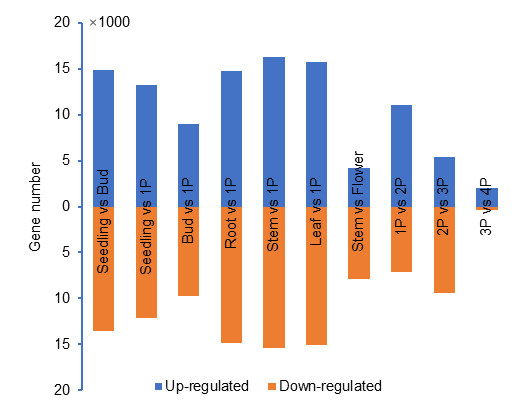
**

**Supplementary Figure S6 The number of DEGs between different tissues in RNA-seq.** DEGs between two tissues with a parameter of |log2Ratio| ≥ 1 and *q* (corrected *p*-value) < 0.05. 1P, 2P, 3P, and 4P represent one-, two-, three-, and four-year-old pseudobulbs, respectively.

**
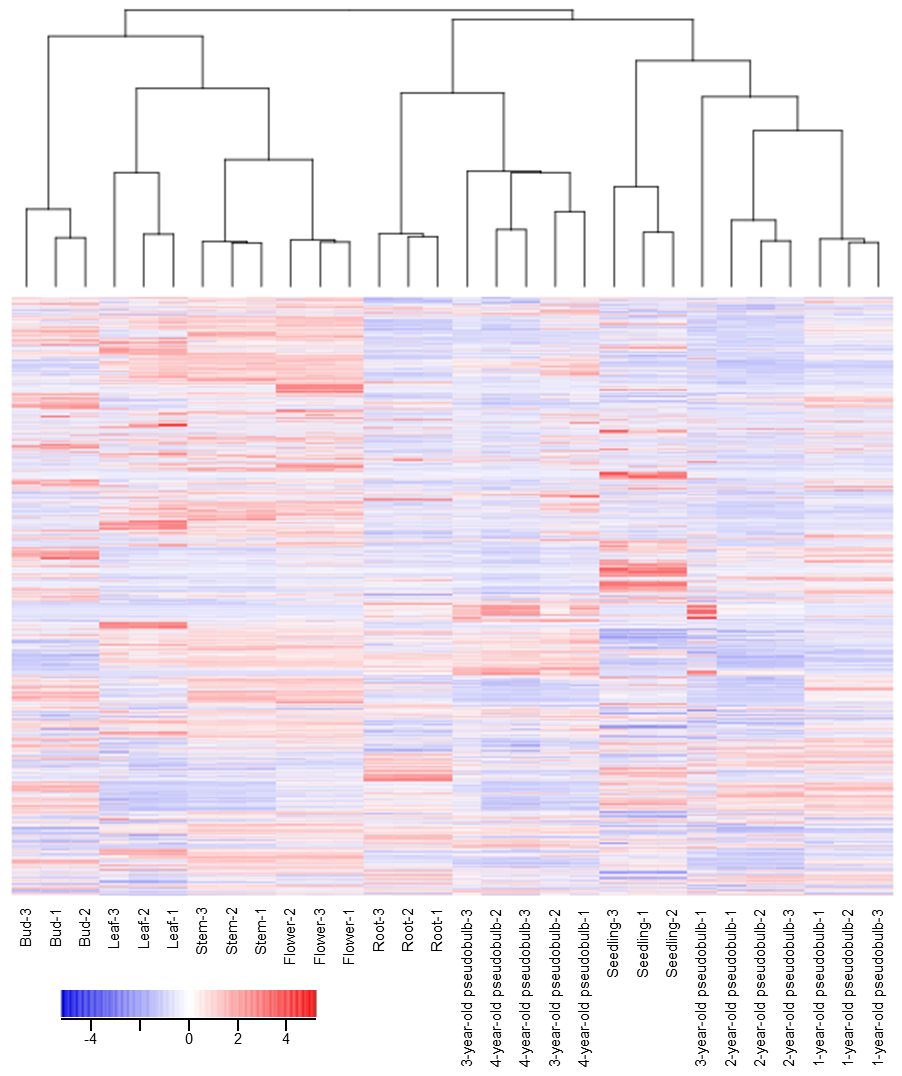
**

**Supplementary Figure S7 Hierarchical cluster of differentially expressed genes.**


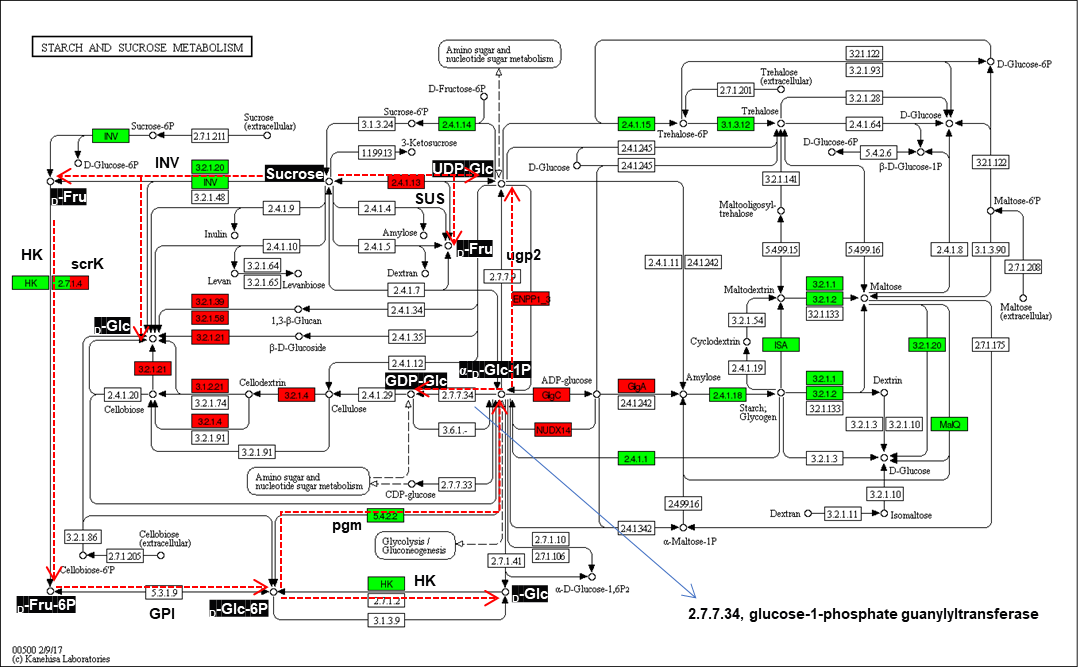


**Supplementary Figure S8 DEGs between multiple tissues and one-year-old pseudobulb matched to the starch and sucrose metabolism (map00500) of the KEGG metabolic pathway.** Red dashed lines indicate possible metabolic fluxes from sucrose to monosaccharides. Red and green box represent the up- and down-regulated genes, respectively. The numbers in boxes represent the EC number of the enzyme.

**
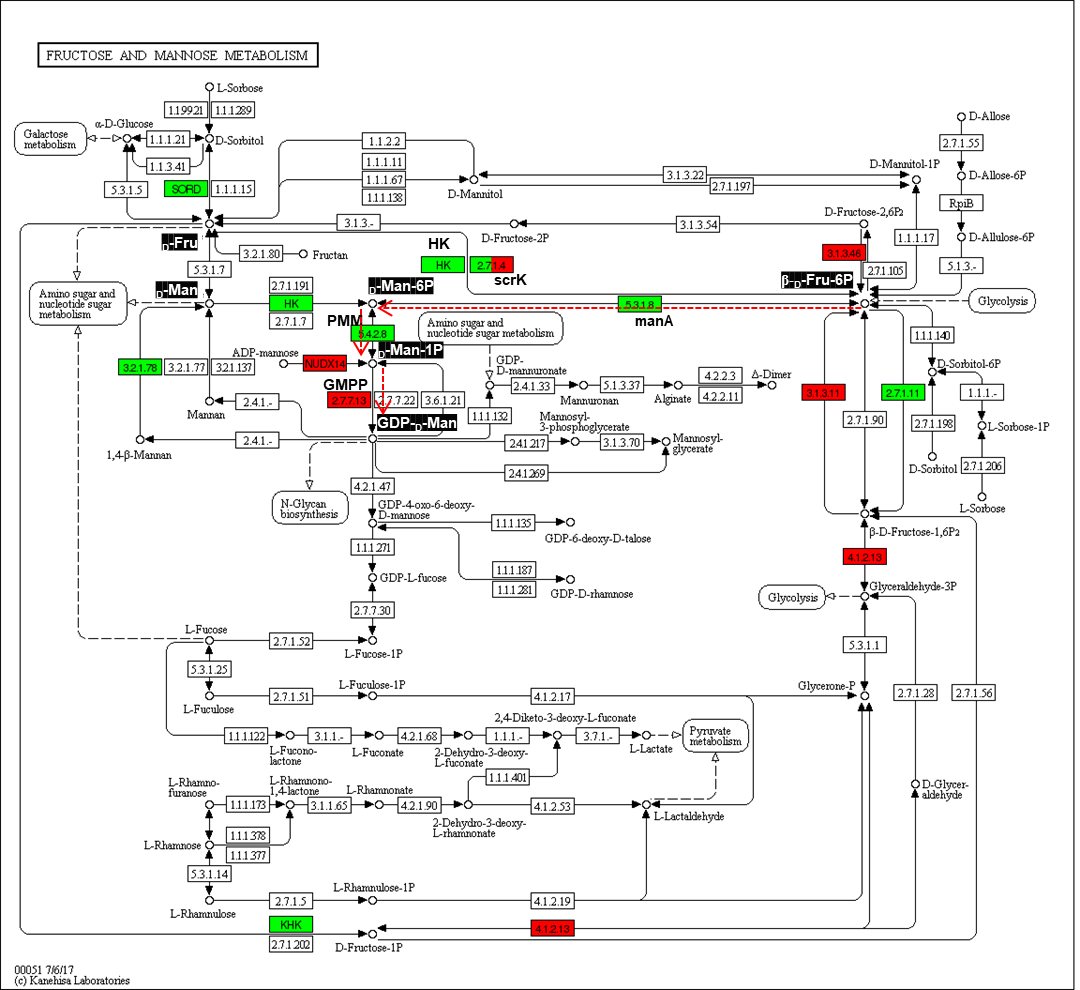
**

**Supplementary Figure S9 DEGs between buds and one-year-old pseudobulb matched to the fructose and mannose metabolism (map00051) of the KEGG metabolic pathway.** Red dashed lines indicate possible metabolic fluxes from _D_-Fru-6P to GDP-_D_-Man. The red and green boxs represent the up- and down-regulated genes, respectively. The numbers in boxes represent the EC number of the enzyme.


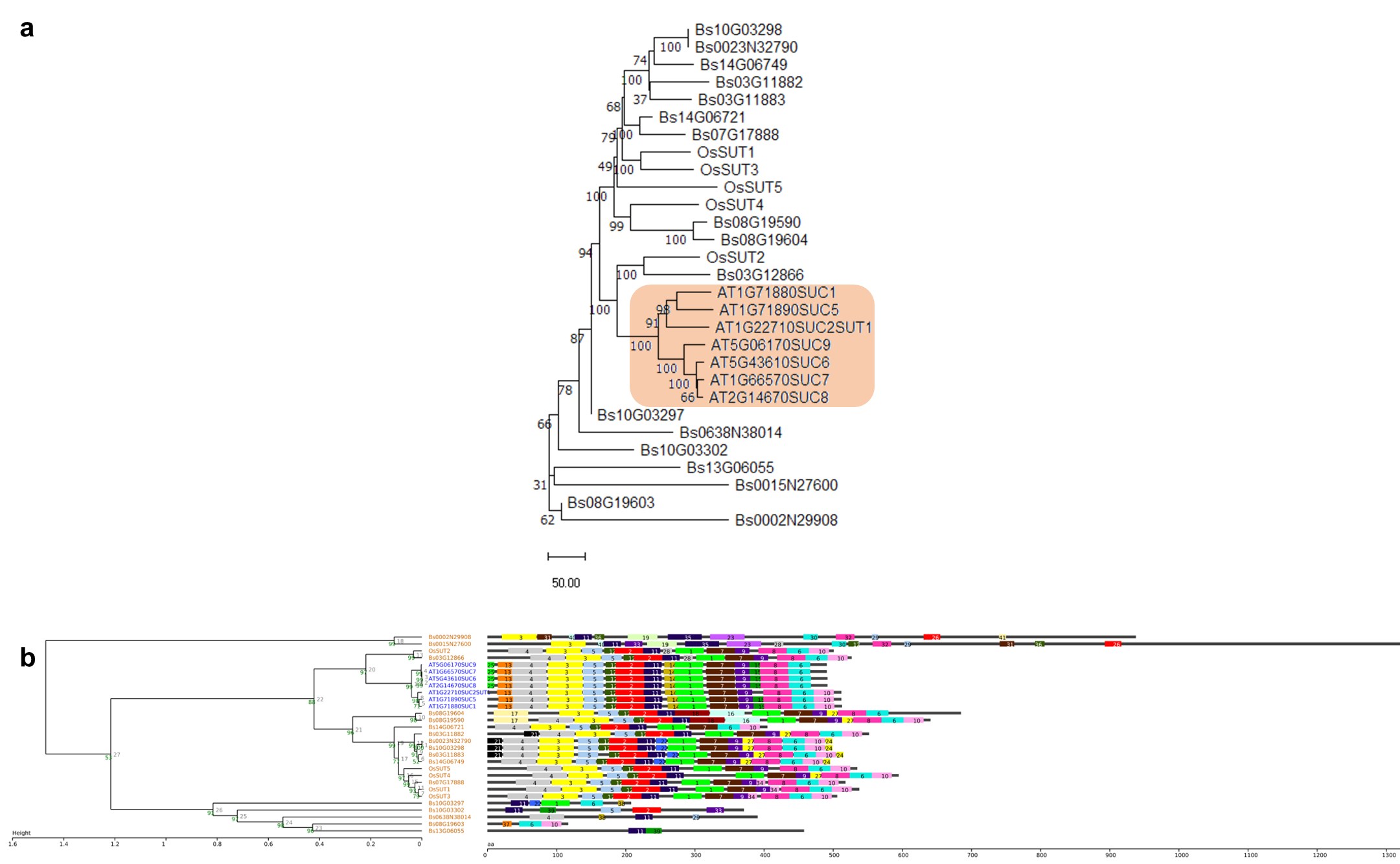


**Supplementary Figure S10 Phylogenetic relationship of seventeen *B. striata* (Bs) SUC, five rice *Oryza sativa* (Os) SUT homologs, and seven Arabidopsis (AT) SUC members.** (**a**) Phylogenetic analysis of the SUC family of *B. striata*, *Oryza sativa* from NCBI (https://www.ncbi.nlm.nih.gov/), and *Arabidopsis thaliana* from TAIR (https://www.arabidopsis.org/Blast/index.jsp). OsSUT1 (NP_001388983.1), OsSUT2 (NP_001391701.1), OsSUT3 (XP_015613774.1), OsSUT4 (NP_001388943.1), and OsSUT5 (NP_001388813.1). AT5G13170 (AtSWEET15) is known as SAG29 (senescence-associated gene 29), AT5G40260 (AtSWEET8) and AT5G50800 (AtSWEET13) are known as AtRPG1 (ruptured pollen grain) and AtRGP2, respectively, and AT5G62850 (AtSWEET5) is known as VEX1 (vegetative cell expressed 1). The amino acid sequence was aligned by ClustalW and the neighbor-joining phylogenetic tree was inferred with MEGA11 from 1000 replicates. (**b**) SALAD analysis of BsSWEETs, OsSUTs, and AtSWEETs. Green number indicates bootstrap value; gray number denotes node number. Right: Motif structure comparison using interactive SALAD analysis (http://salad.dna.affrc.go.jp/CGViewer/en/cgv_upload.html).


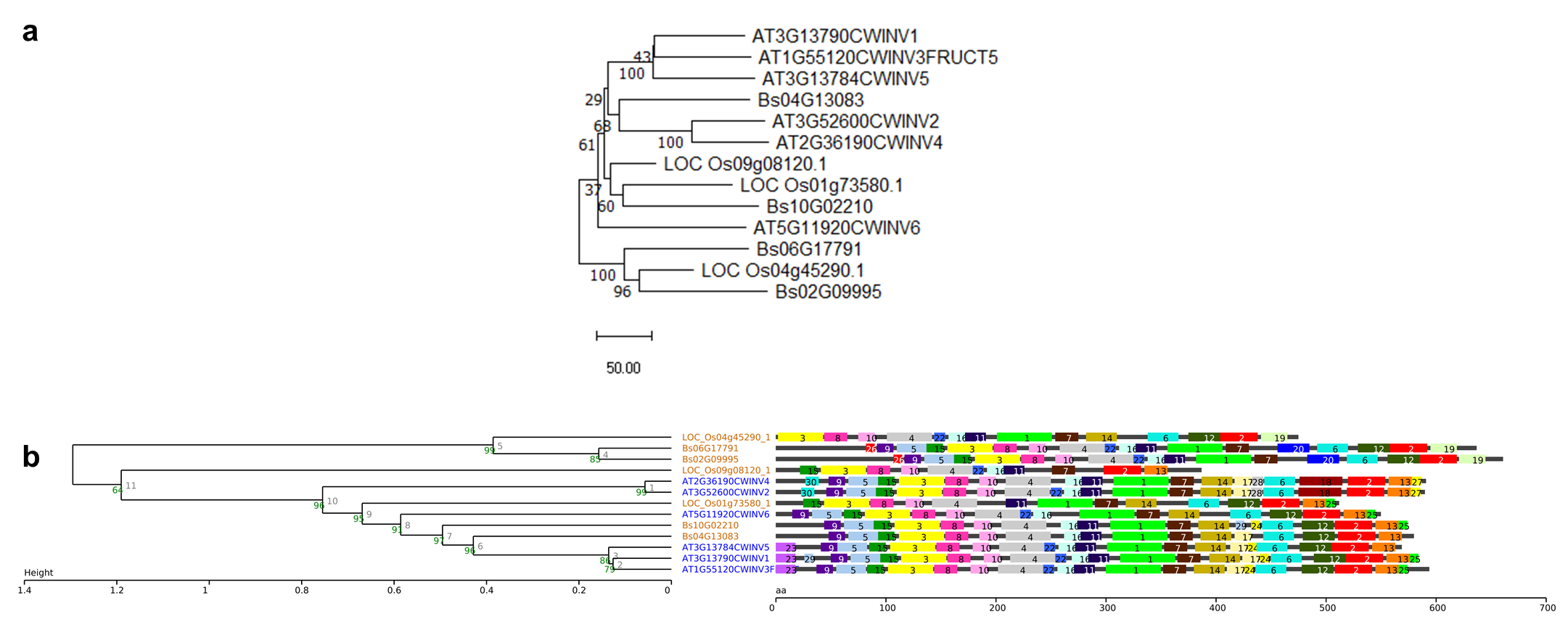


**Supplementary Figure S11 Phylogenetic relationship of four *B. striata* (Bs) INVs, three rice *Oryza sativa* (Os) homologs, and six Arabidopsis INV members (CWINV, cell wall invertase).** (**a**) Phylogenetic analysis of the INV (invertase) family, also known as sacA (beta-fructofuranosidase), of *B. striata*, *Oryza sativa* from the China Rice Data Center (www.ricedata.com), and *A. thaliana* from TAIR by MEGA11 with the same method as **Supplementary Figure S10**. AT1G55120 (AtCWINV3) is known as FRUCT5 (beta-fructofuranosidase 5). (**b**) SALAD analysis of BsINVs, OsINVs, and AtCWINVs.


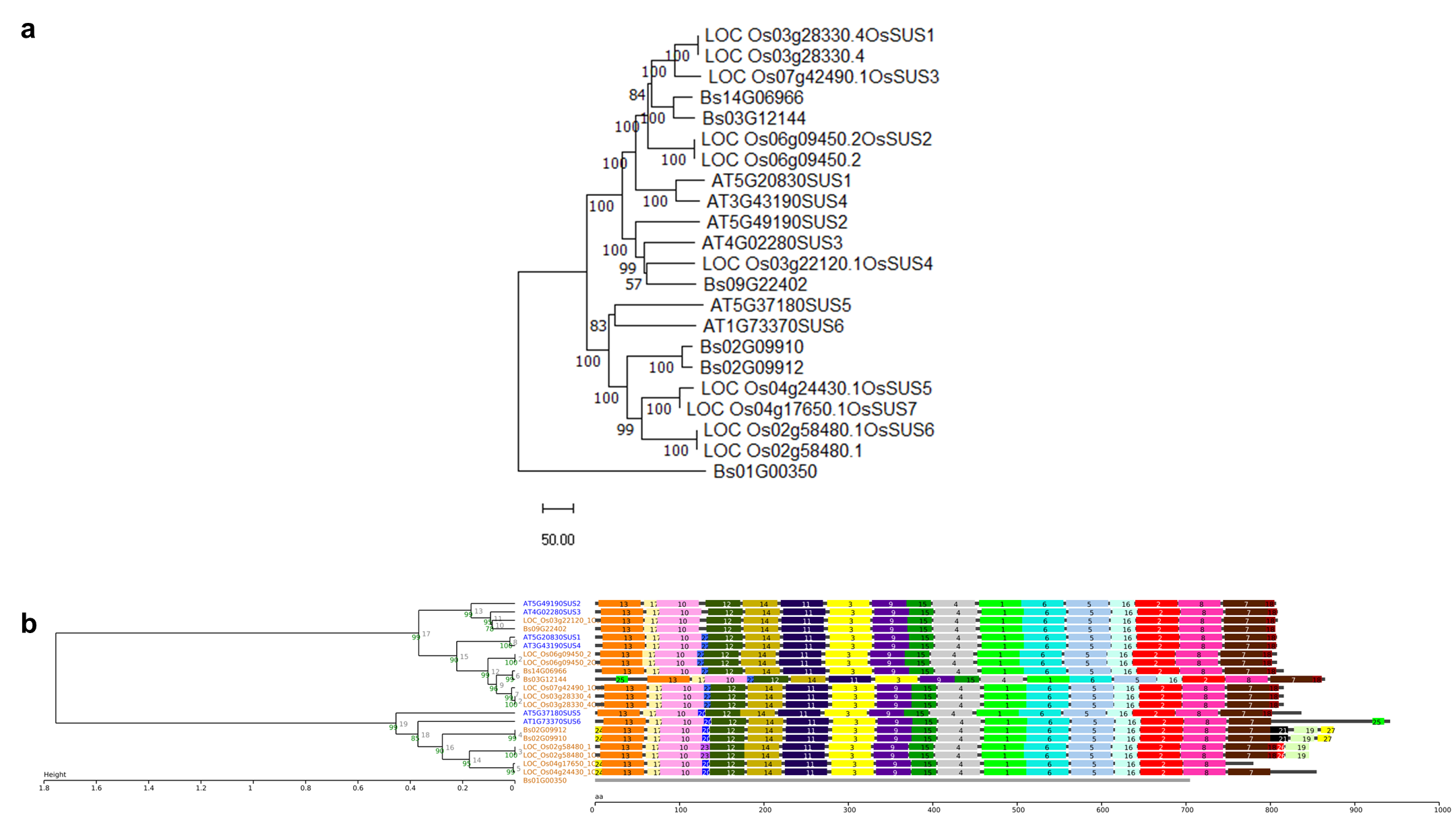


**Supplementary Figure S12 Phylogenetic relationship of six *B. striata* (Bs) SUSs, ten rice *Oryza sativa* (Os) homologs, and six Arabidopsis SUS members.** (**a**) Phylogenetic analysis of the SUS family of *B. striata*, *O. sativa*, and *A. thaliana* by MEGA11 with the same method as **Supplementary Figure S10**. (**b**) SALAD analysis of BsSUSs, OsSUSs, and AtSUSs.


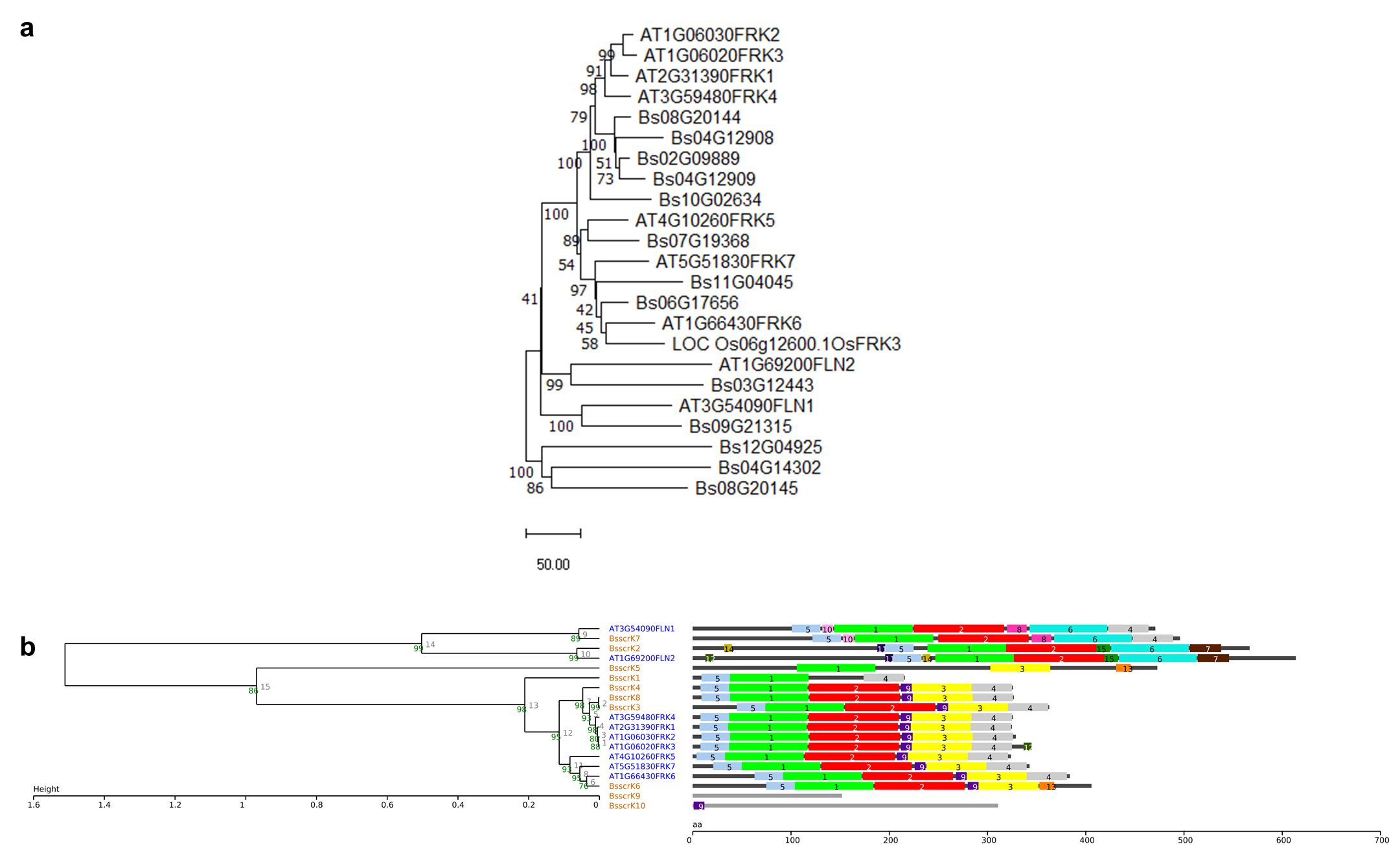


**Supplementary Figure S13 Phylogenetic relationship of thirteen *B. striata* (Bs) scrKs (fructokinase, FRK), one *Oryza sativa* (Os) homolog, and Arabidopsis seven FRK and two FLN (fructokinase-like) members.** (**a**) Phylogenetic analysis of the scrK family of *B. striata*, *O. sativa*, and *A. thaliana* by MEGA11 with the same method as **Supplementary Figure S10**. (**b**) SALAD analysis of BsscrKs, OsFRK, and AtFRKs.


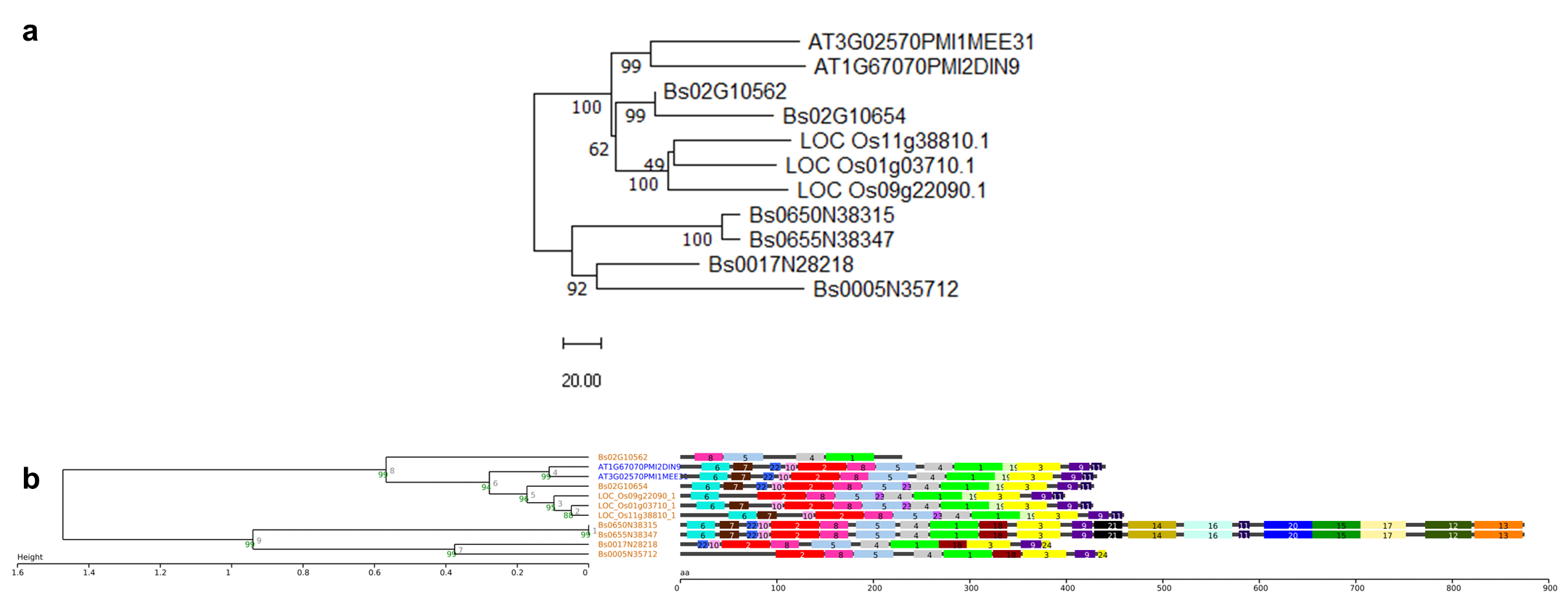


**Supplementary Figure S14 Phylogenetic relationship of six *B. striata* (Bs) mannose-6-phosphate isomerase (manA), three rice *Oryza sativa* (Os) homologs, and two Arabidopsis (AT) phosphomannose isomerase (PMI) members.** (**a**) Phylogenetic analysis of the manA family of *B. striata*, *O. sativa*, and *A. thaliana* by MEGA11 with the same method as **Supplementary Figure S10**. AT3G02570 (PMI1 or maternal effect embryo arrest 31, MEE31), AT1G67070 (AtPMI2 or DIN9, dark inducible 9). (**b**) SALAD analysis of BsmanAs, OsmanAs, and AtPMIs.


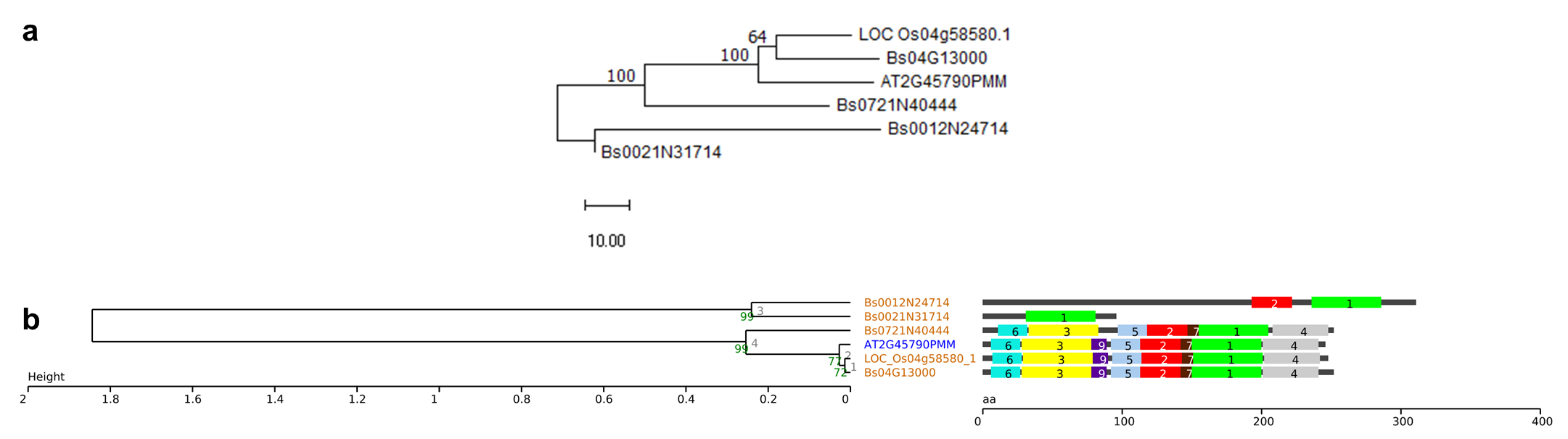


**Supplementary Figure S15 Phylogenetic relationship of four *B. striata* (Bs) phosphomannomutase (PMM), one rice *Oryza sativa* (Os) homolog, and one Arabidopsis (AT) PMM member.** (**a**) Phylogenetic analysis of the PMM family of *B. striata*, *O. sativa*, and *A. thaliana* by MEGA11 with the same method as **Supplementary Figure S10**. (**b**) SALAD analysis of BsPMMs, OsPMM, and AtPMM.


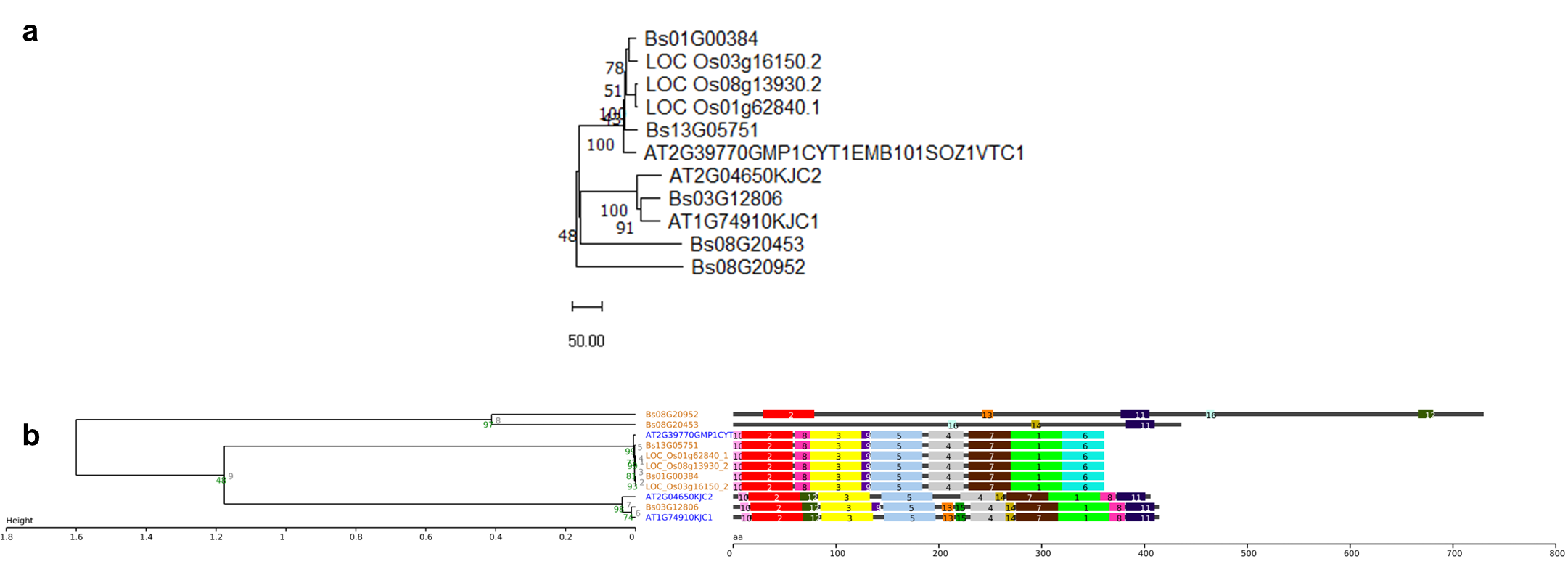


**Supplementary Figure S16 Phylogenetic relationship of five *B. striata* (Bs) GMPP (GDP-mannose pyrophosphorylase), three rice *Oryza sativa* (Os) homologs, and three Arabidopsis (AT) GMP members.** (**a**) Phylogenetic analysis of the GMPP family of *B. striata*, *O. sativa*, and *A. thaliana* by MEGA11 with the same method as **Supplementary Figure S10**. AtGMP1 (AT2G39770) is also known as AtCYT1 (CYTOKINESIS DEFECTIVE 1), EMB101 (EMBRYO DEFECTIVE 101), SOZ1 (SENSITIVE TO OZONE 1), and VTC1 (VITAMIN C DEFECTIVE 1). KJC1 (KONJAC1, AT1G74910) and KJC2 (AT2G04650) are similar to sugar pyrophosphorylases but have an insertion of 2 AA in the pyrophosphorylase consensus motif that is highly conserved in GMPPs. It lacks GDP-mannose pyrophosphorylase activity but can simulate the GDP-mannose pyrophosphorylase activity of VTC1. (**b**) SALAD analysis of BsGMPPs, OsGMPPs, and AtGMPPs.


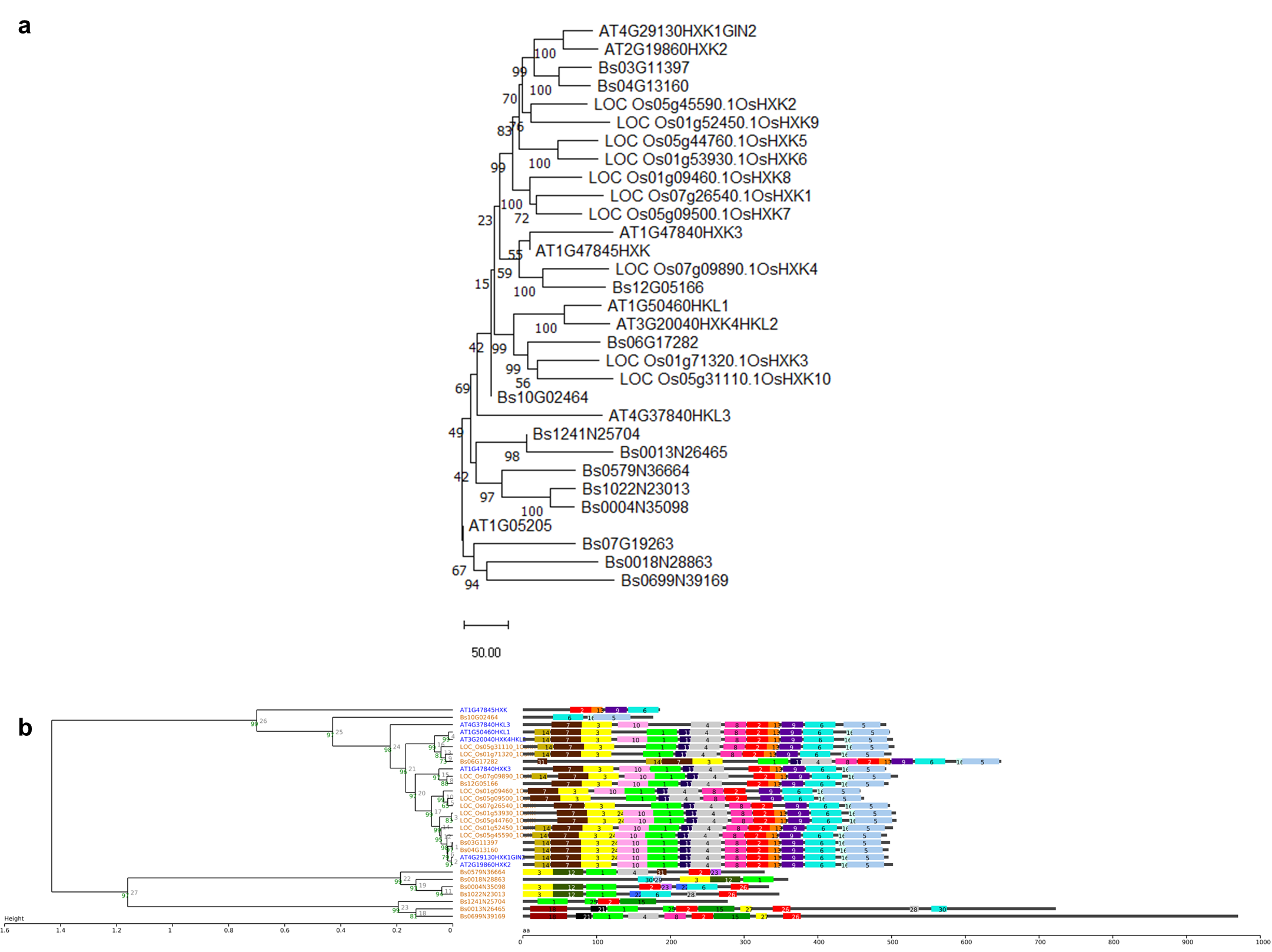


**Supplementary Figure S17 Phylogenetic relationship of thirteen *B. striata* (Bs) HK (hexokinase), ten rice *Oryza sativa* (Os) homologs, and eight Arabidopsis HXK and HKL (HEXOKINASE-LIKE) members.** (**a**) Phylogenetic analysis of the HK family of *B. striata*, *O. sativa*, and *A. thaliana* by MEGA11 with the same method as **Supplementary Figure S10**. AtHXK1, also called GIN2, GLUCOSE INSENSITIVE 2. HKL1 (AT1G76030, AtVAB1, V-ATPASE B SUBUNIT 1), one of three genes encoding the vacuolar ATP synthase subunit B1. This subunit was shown to interact with the gene product of hexokinase 1 (ATHXK1). (**b**) SALAD analysis of BsHKs, OsHKs, and AtHXKs.


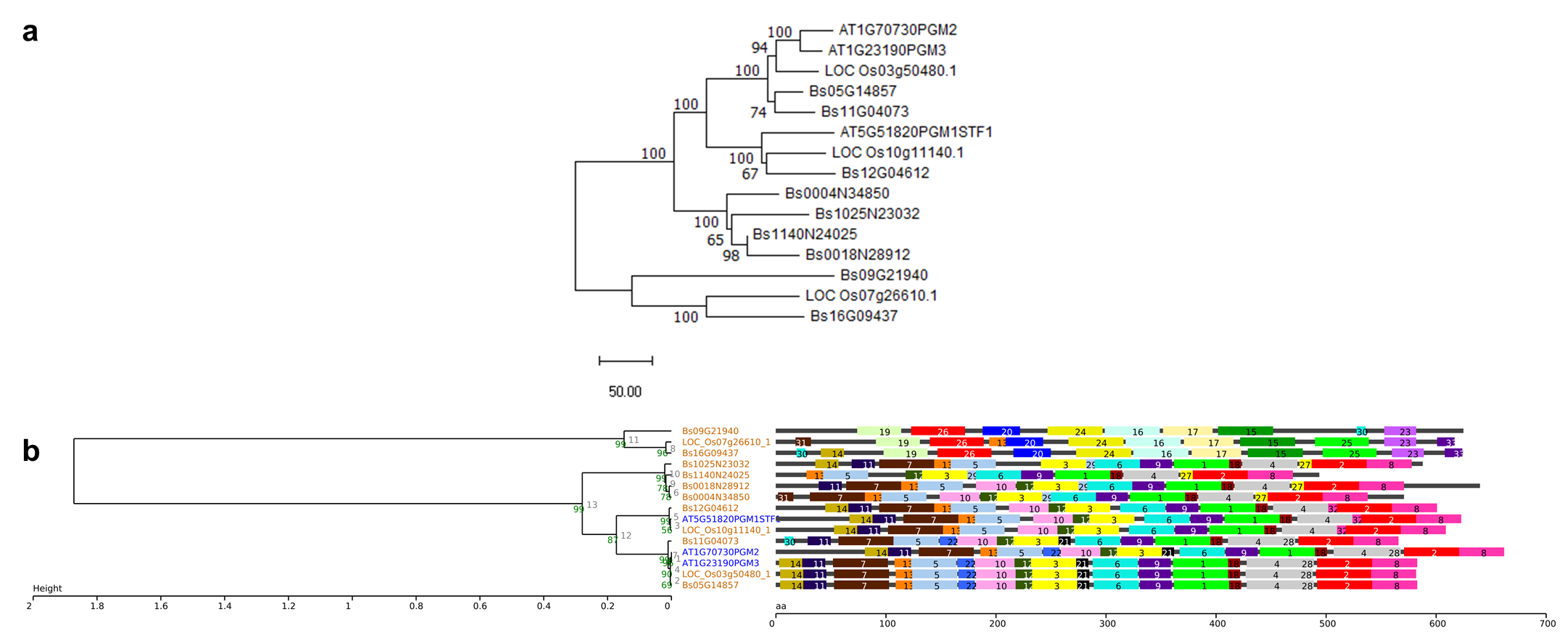


**Supplementary Figure S18 Phylogenetic relationship of seven *B. striata* (Bs) pgm (phosphoglucomutase), three rice *Oryza sativa* (Os) homologs, and three Arabidopsis pgm members.** (**a**) Phylogenetic analysis of the pgm family of *B. striata*, *O. sativa*, and *A. thaliana* by MEGA11 with the same method as **Supplementary Figure S10**. AtPGM1 (AT5G51820) also the name of AtSTF1 (STARCH-FREE 1). (**b**) SALAD analysis of Bspgms, Ospgms, and Atpgms.


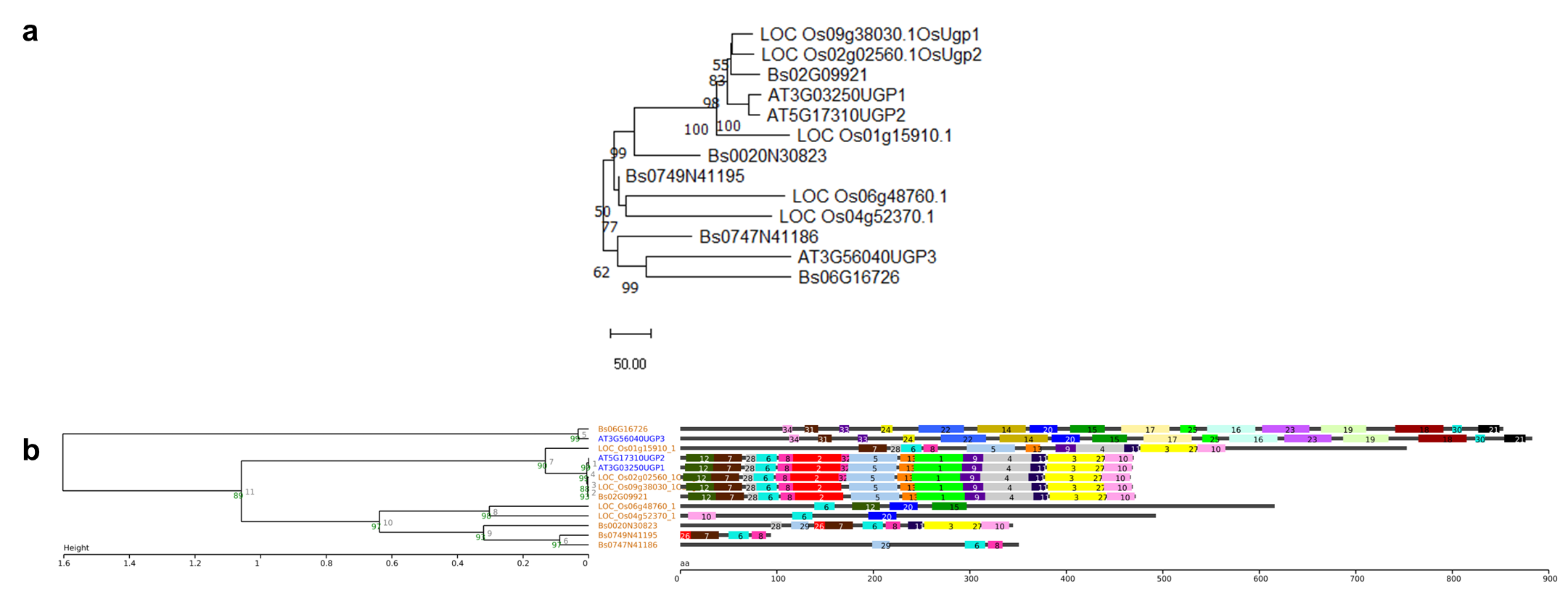


**Supplementary Figure S19 Phylogenetic relationship of three *B. striata* (Bs) UGP2 (UDP-GLUCOSE PYROPHOSPHORYLASE, or UTP--glucose-1-phosphate uridylyltransferase), five rice *Oryza sativa* (Os) homologs, and three Arabidopsis UGP members.** (**a**) Phylogenetic analysis of the UGP2 family of *B. striata*, *O. sativa*, and *A. thaliana* by MEGA11 with the same method as **Supplementary Figure S10**. (**b**) SALAD analysis of BsUGP2s, OsUGP2s, and AtUGPs.


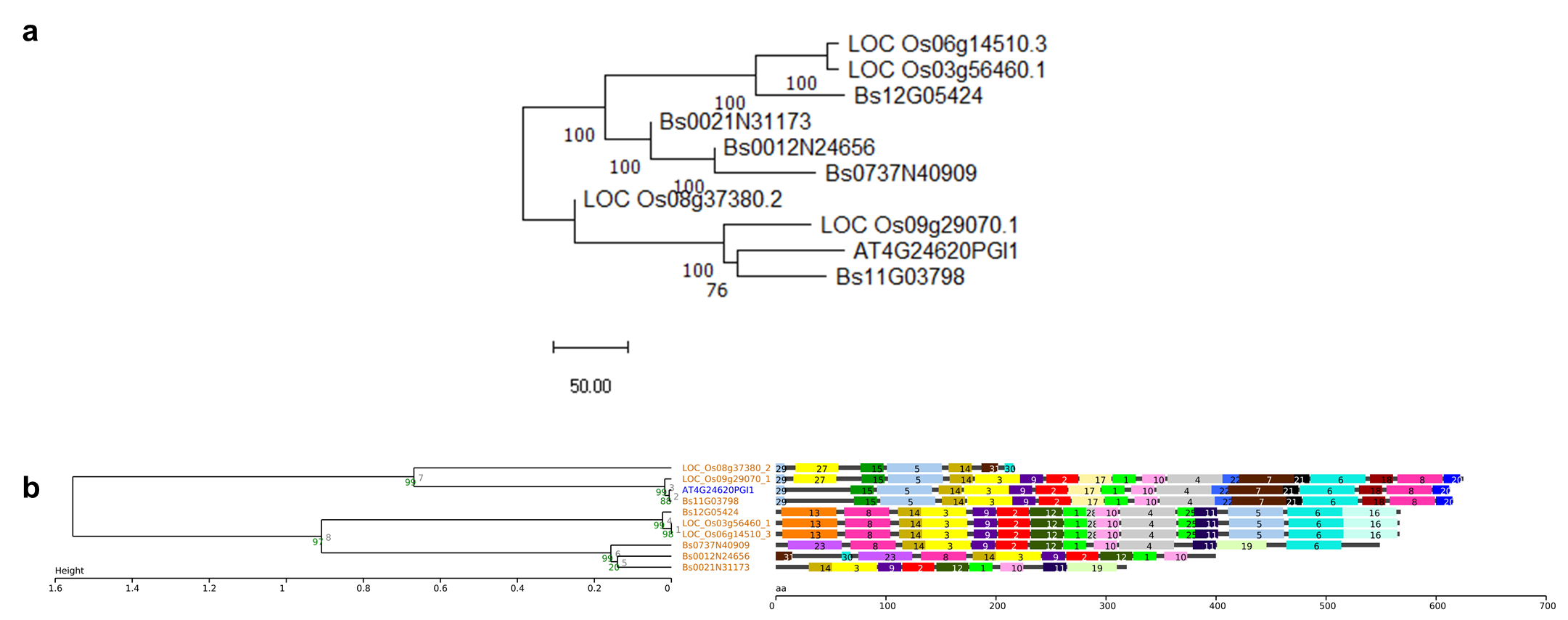


**Supplementary Figure S20 Phylogenetic relationship of four *B. striata* (Bs) GPI (glucose-6-phosphate isomerase), four rice *Oryza sativa* (Os) homologs, and one Arabidopsis PGI (PHOSPHOGLUCOSE ISOMERASE) member.** (**a**) Phylogenetic analysis of GPI family of *B. striata*, *O. sativa*, and *A. thaliana* by MEGA11 with the same method as **Supplementary Figure S10**. (**b**) SALAD analysis of BsGPIs, PsGPIs, and AtPGI1.


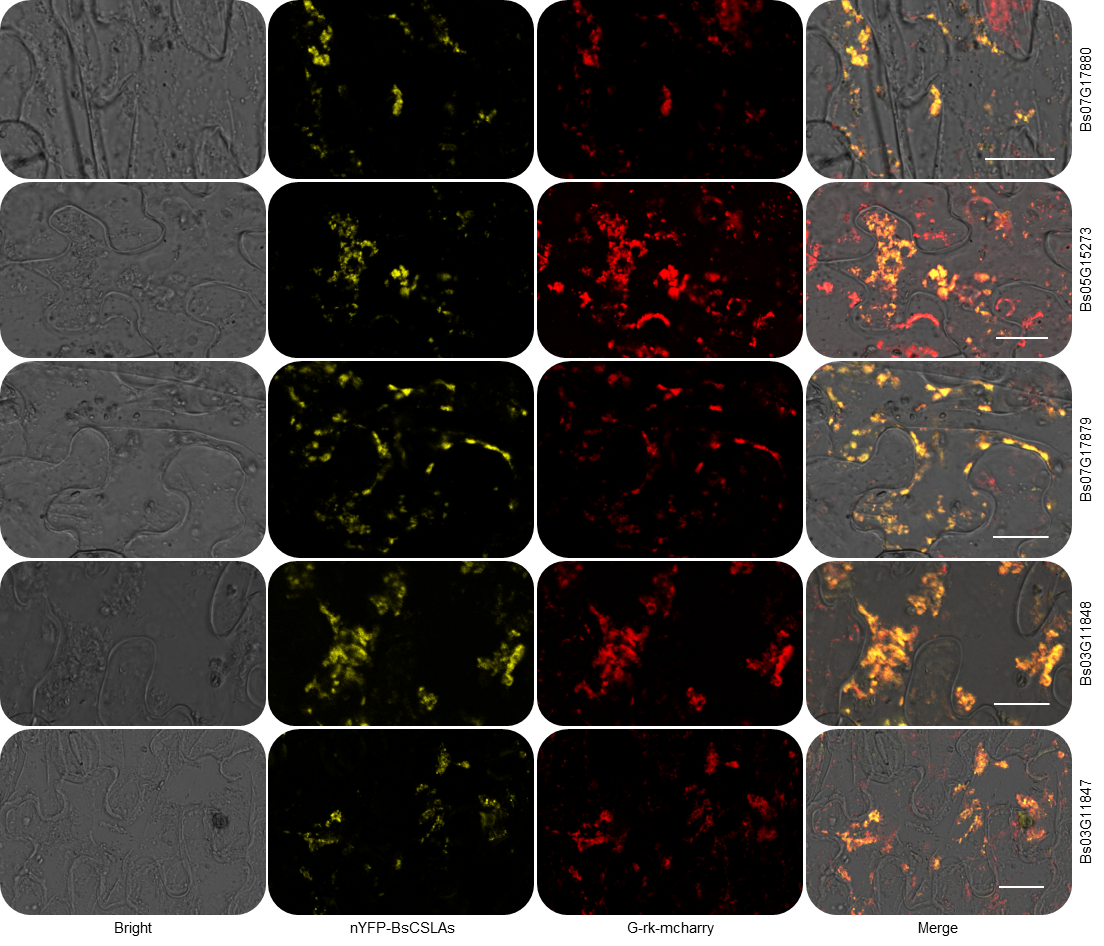


**Supplementary Figure S21 Subcellular localization assay of five cellulose synthase-like family A (CSLA) members.** Bars = 20 µm.


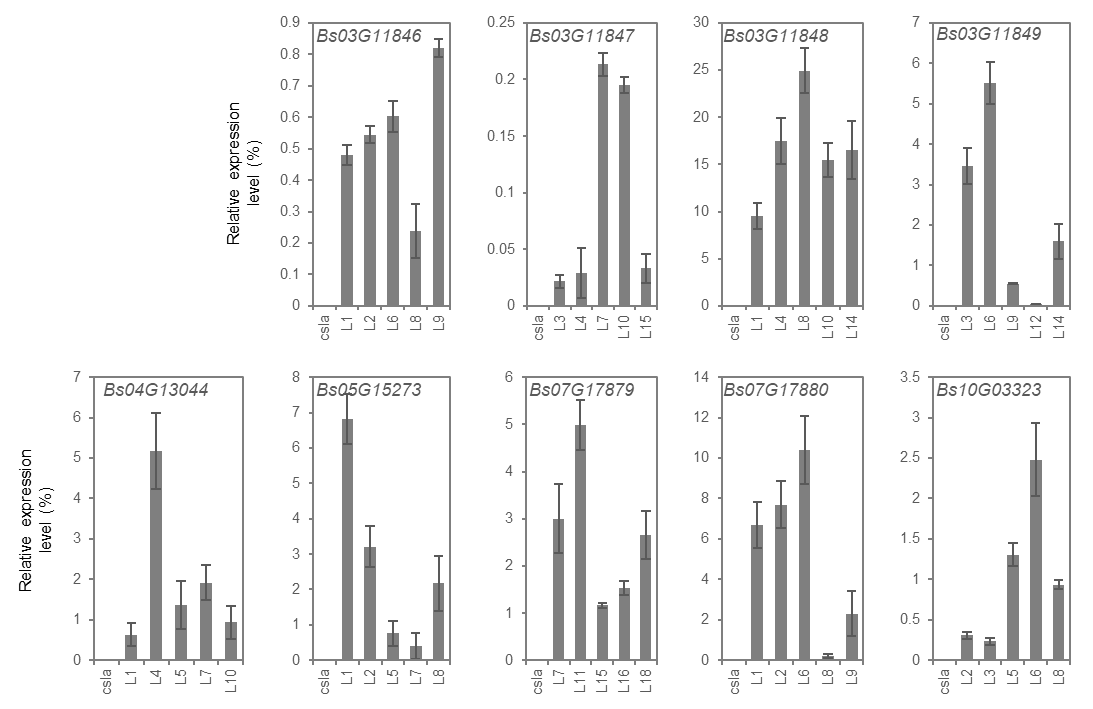


**Supplementary Figure S22 Gene expression analysis in the *csla2-1csla3-2csla9-1* triple mutant (*csla*) and each of the five respective independent complemented lines of BsCSLAs.** Each BsCSLA complementary transgenic had more than ten independent lines. Total RNA was isolated from the 10-cm stem at the bottom of the 8-week-old inflorescence of the T1 generation of five independent lines for qRT-PCR characterization. The housekeeping gene ACT2 was used as a reference gene for normalization. The values represent the mean ± SD (n = 3).


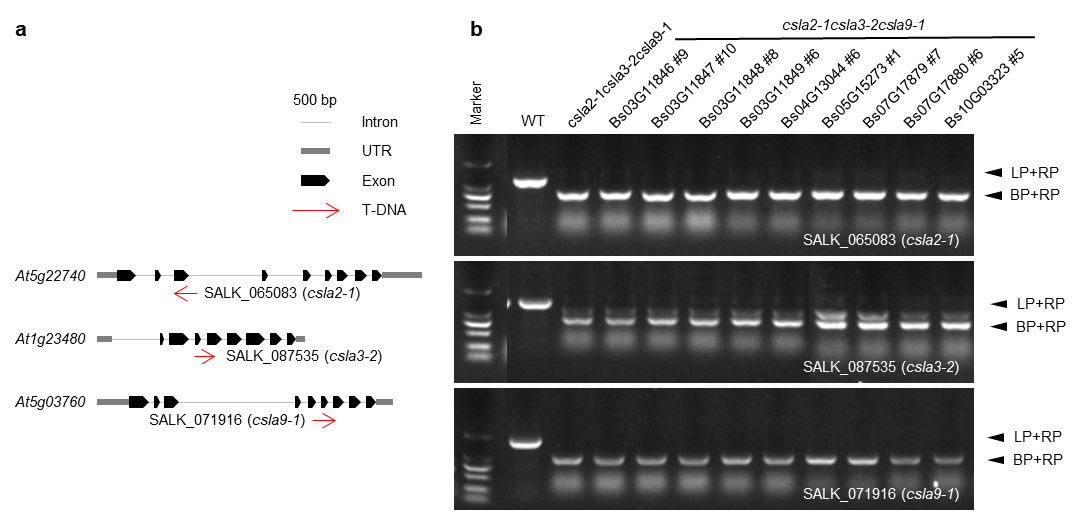


**Supplementary Figure S23 Genotyping of *csla2-1csla3-2csla9-1* triple mutant and BsCSLAs complemented lines.**


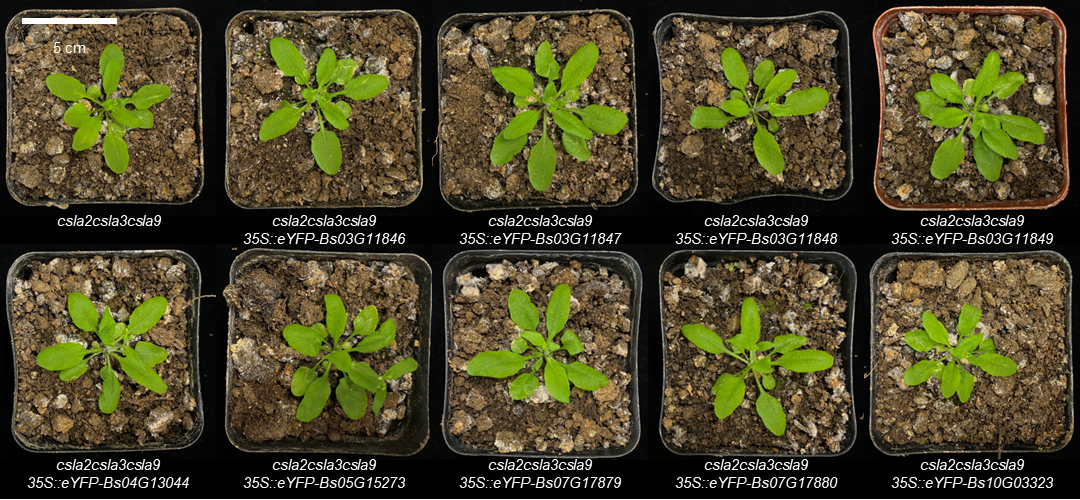


**Supplementary Figure S24 4-week-old plants of the *cesa2csla3csla9* triple mutant and the complement transgenic lines of *BsCSLA*s.** Bars = 5 cm.


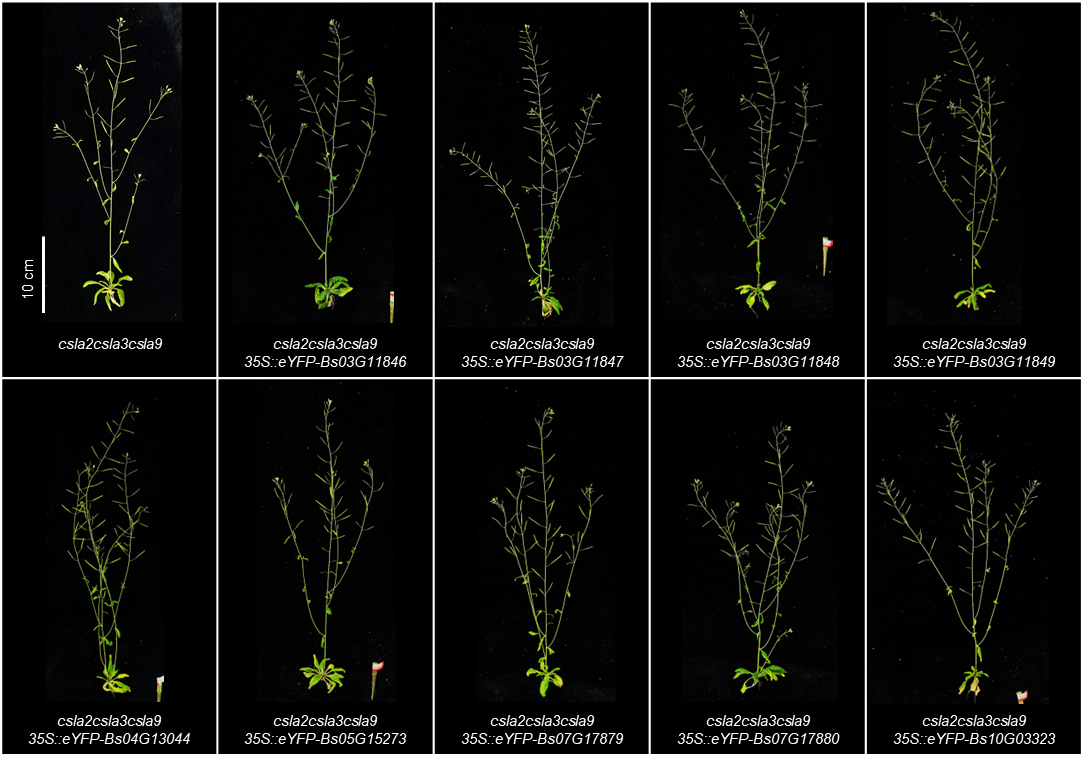


**Supplementary Figure S25 8-week-old plants of the *cesa2csla3csla9* triple mutant and the complement transgenic lines of *BsCSLA*s.** Bars = 10 cm.


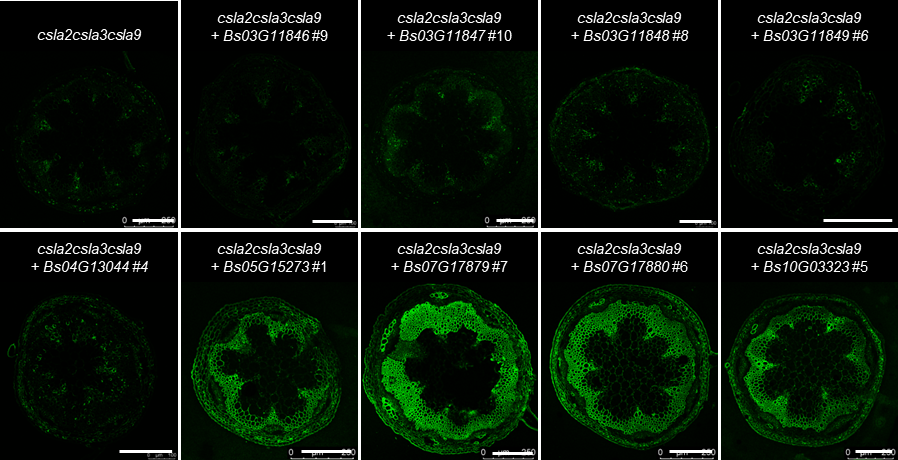


**Supplementary Figure S26 Complement assay of Arabidopsis stem glucomannan biosynthesis.** Bars = 200 µm.


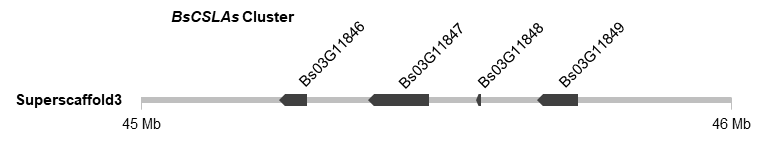


**Supplementary Figure S27 *BsCSLA*s gene cluster in superscaffold3.**


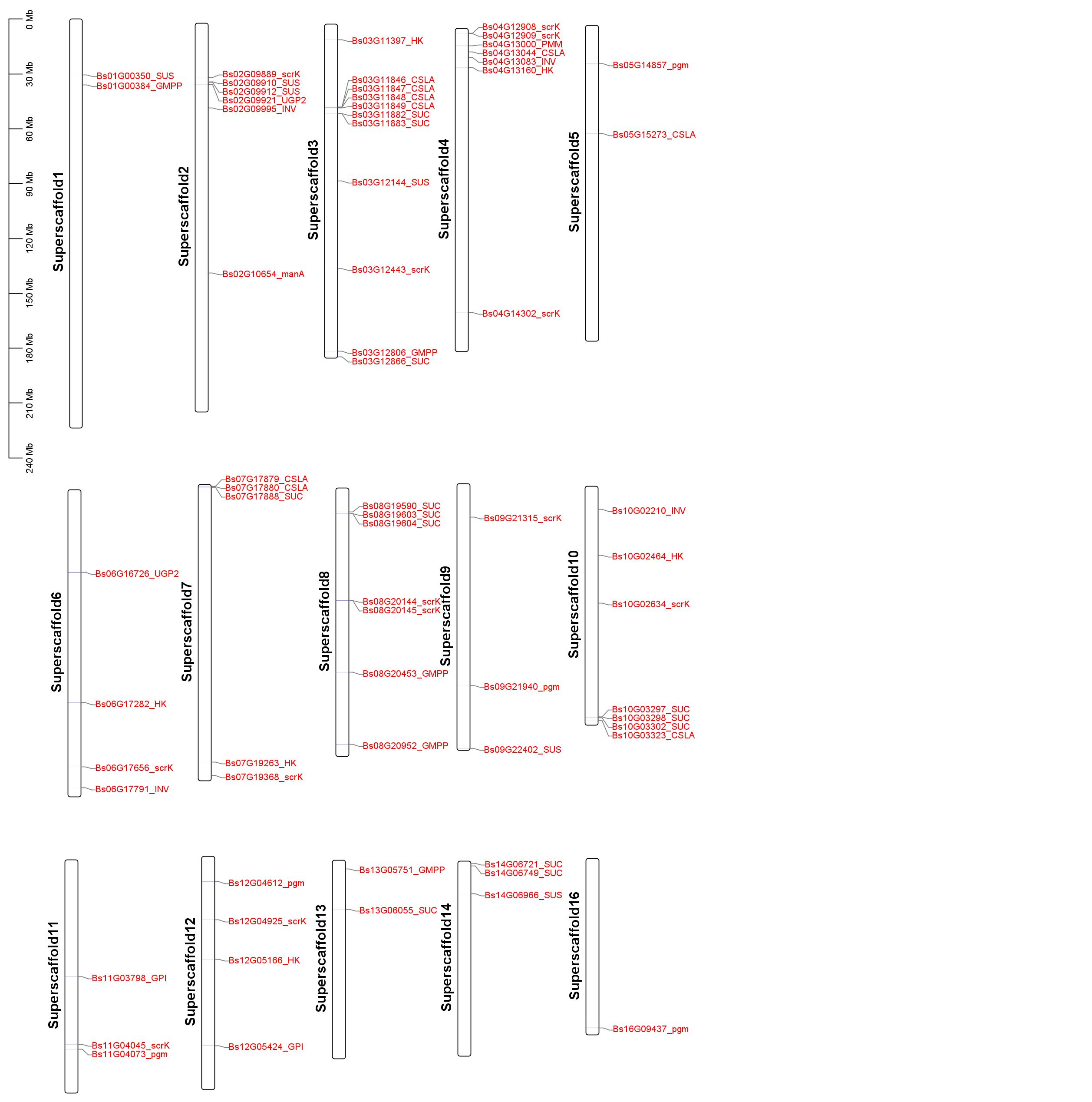


**Supplementary Figure S28 Distribution of BSP-related genes on *B. striata* chromosomes.**

**Supplementary Table S1 Content of monosaccharides corresponding to three unknown peaks**

| **Tissue** | **Monosaccharide composition (mg/g)** | | |
| --- | --- | --- | --- |
|  | Un1 | Un2 | Un3 |
| Root | 8.42±0.31 | 1.28±0.86 | 1.62±0.22 |
| Leaf | 1.92±1.38 | 21.68±5.39 | 0.97±0.05 |
| One-year-old pseudobulb | 8.22±1.38 | ns | ns |
| Two-year-old pseudobulb | 7.08±1.96 | ns | ns |
| Three-year-old pseudobulb | 8.23±0.84 | ns | ns |
| Four-year-old pseudobulb | 7.66±0.49 | ns | ns |

Data shown are mean±SD (n = 3). Un1, Un2 and Un3 represent unknown peaks in **Supplementary Figure S3**.

**Supplementary Table S2 Significant enrichment KEGG pathway of DEGs in different groups**

| **Group** | **Pathway Name** | **Map** | **Count1** | **Count2** | **Count3** | **Count4** | ***p*** | ***q*** | **Up count** | **Down count** |
| --- | --- | --- | --- | --- | --- | --- | --- | --- | --- | --- |
| Seedling/1P | Phenylpropanoid biosynthesis | map00940 | 235 | 187 | 6115 | 10329 | 2.13E-14 | 2.94E-12 | 142 | 93 |
|  | Starch and sucrose metabolism | map00500 | 278 | 300 | 6072 | 10216 | 1.21E-07 | 8.34E-06 | 157 | 121 |
|  | Cyanoamino acid metabolism | map00460 | 138 | 134 | 6212 | 10382 | 6.47E-06 | 0.000298 | 83 | 55 |
|  | alpha-Linolenic acid metabolism | map00592 | 76 | 71 | 6274 | 10445 | 0.000345 | 0.009409 | 50 | 26 |
|  | Plant hormone signal transduction | map04075 | 269 | 339 | 6081 | 10177 | 0.000409 | 0.009409 | 161 | 108 |
|  | MAPK signaling pathway - plant | map04016 | 184 | 219 | 6166 | 10297 | 0.000532 | 0.010483 | 113 | 71 |
|  | Glutathione metabolism | map00480 | 114 | 126 | 6236 | 10390 | 0.001074 | 0.018524 | 71 | 43 |
|  | Inositol phosphate metabolism | map00562 | 109 | 122 | 6241 | 10394 | 0.001802 | 0.027632 | 66 | 43 |
|  | Cutin, suberine and wax biosynthesis | map00073 | 39 | 32 | 6311 | 10484 | 0.002242 | 0.02813 | 26 | 13 |
|  | Glycosphingolipid biosynthesis - globo and isoglobo series | map00603 | 20 | 11 | 6330 | 10505 | 0.002175 | 0.02813 | 12 | 8 |
| Bud/1P | Plant hormone signal transduction | map04075 | 227 | 381 | 3729 | 12529 | 3.79E-15 | 5.23E-13 | 82 | 145 |
|  | Photosynthesis | map00195 | 69 | 55 | 3887 | 12855 | 1.02E-14 | 7.02E-13 | 67 | 2 |
|  | MAPK signaling pathway - plant | map04016 | 159 | 244 | 3797 | 12666 | 3.23E-13 | 1.11E-11 | 48 | 111 |
|  | Phenylpropanoid biosynthesis | map00940 | 165 | 257 | 3791 | 12653 | 2.84E-13 | 1.11E-11 | 86 | 79 |
|  | Fatty acid elongation | map00062 | 59 | 62 | 3897 | 12848 | 1.03E-09 | 2.83E-08 | 28 | 31 |
|  | alpha-Linolenic acid metabolism | map00592 | 64 | 83 | 3892 | 12827 | 5.58E-08 | 1.22E-06 | 6 | 58 |
|  | Starch and sucrose metabolism | map00500 | 190 | 388 | 3766 | 12522 | 1.02E-07 | 1.76E-06 | 84 | 106 |
|  | Plant-pathogen interaction | map04626 | 150 | 303 | 3806 | 12607 | 1.40E-06 | 1.93E-05 | 34 | 116 |
|  | Flavonoid biosynthesis | map00941 | 23 | 19 | 3933 | 12891 | 1.16E-05 | 0.000146 | 7 | 16 |
|  | Photosynthesis - antenna proteins | map00196 | 25 | 29 | 3931 | 12881 | 0.00019 | 0.002189 | 22 | 3 |
|  | Stilbenoid, diarylheptanoid and gingerol biosynthesis | map00945 | 11 | 6 | 3945 | 12904 | 0.000343 | 0.003637 | 0 | 11 |
|  | Linoleic acid metabolism | map00591 | 25 | 32 | 3931 | 12878 | 0.000531 | 0.005235 | 2 | 23 |
|  | Sphingolipid metabolism | map00600 | 55 | 109 | 3901 | 12801 | 0.002075 | 0.01909 | 10 | 45 |
|  | Ubiquinone and other terpenoid-quinone biosynthesis | map00130 | 48 | 92 | 3908 | 12818 | 0.00233 | 0.020099 | 37 | 11 |

**Supplementary Table S2 Continued.**

| **Group** | **Pathway Name** | **Map** | **Count1** | **Count2** | **Count3** | **Count4** | ***p*** | ***q*** | **Up count** | **Down count** |
| --- | --- | --- | --- | --- | --- | --- | --- | --- | --- | --- |
| Bud/1P | Carbon fixation in photosynthetic organisms | map00710 | 80 | 179 | 3876 | 12731 | 0.003483 | 0.028276 | 46 | 34 |
|  | Diterpenoid biosynthesis | map00904 | 17 | 22 | 3939 | 12888 | 0.004318 | 0.033108 | 1 | 16 |
|  | Carotenoid biosynthesis | map00906 | 39 | 75 | 3917 | 12835 | 0.005905 | 0.042887 | 21 | 18 |
|  | DNA replication | map03030 | 51 | 107 | 3905 | 12803 | 0.006924 | 0.047774 | 47 | 4 |
| Root/1P | Plant hormone signal transduction | map04075 | 308 | 300 | 6379 | 9879 | 1.42E-08 | 1.96E-06 | 168 | 140 |
|  | Phenylpropanoid biosynthesis | map00940 | 216 | 206 | 6471 | 9973 | 7.79E-07 | 5.37E-05 | 130 | 86 |
|  | Starch and sucrose metabolism | map00500 | 284 | 294 | 6403 | 9885 | 1.60E-06 | 7.37E-05 | 124 | 160 |
|  | Plant-pathogen interaction | map04626 | 217 | 236 | 6470 | 9943 | 0.000184 | 0.006334 | 133 | 84 |
|  | Cyanoamino acid metabolism | map00460 | 135 | 137 | 6552 | 10042 | 0.000484 | 0.013345 | 62 | 73 |
|  | Flavonoid biosynthesis | map00941 | 27 | 15 | 6660 | 10164 | 0.001068 | 0.024563 | 18 | 9 |
|  | MAPK signaling pathway - plant | map04016 | 188 | 215 | 6499 | 9964 | 0.002275 | 0.044849 | 106 | 82 |
| Stem/1P | Photosynthesis | map00195 | 86 | 38 | 7154 | 9588 | 2.22E-09 | 3.07E-07 | 78 | 8 |
|  | Phenylpropanoid biosynthesis | map00940 | 236 | 186 | 7004 | 9440 | 3.74E-08 | 2.58E-06 | 126 | 110 |
|  | Plant hormone signal transduction | map04075 | 321 | 287 | 6919 | 9339 | 3.94E-07 | 1.81E-05 | 131 | 190 |
|  | MAPK signaling pathway - plant | map04016 | 211 | 192 | 7029 | 9434 | 7.24E-05 | 0.002497 | 86 | 125 |
|  | Stilbenoid, diarylheptanoid and gingerol biosynthesis | map00945 | 15 | 2 | 7225 | 9624 | 0.00015 | 0.00413 | 0 | 15 |
|  | Flavonoid biosynthesis | map00941 | 28 | 14 | 7212 | 9612 | 0.001615 | 0.037155 | 7 | 21 |
|  | alpha-Linolenic acid metabolism | map00592 | 81 | 66 | 7159 | 9560 | 0.001895 | 0.037356 | 15 | 66 |
| Leaf/1P | Photosynthesis | map00195 | 91 | 33 | 6896 | 9846 | 4.53E-13 | 6.26E-11 | 85 | 6 |
|  | Porphyrin and chlorophyll metabolism | map00860 | 119 | 67 | 6868 | 9812 | 3.87E-10 | 1.78E-08 | 101 | 18 |
|  | Carbon fixation in photosynthetic organisms | map00710 | 148 | 111 | 6839 | 9768 | 2.06E-07 | 5.68E-06 | 101 | 47 |
|  | Pentose phosphate pathway | map00030 | 121 | 85 | 6866 | 9794 | 3.58E-07 | 8.24E-06 | 69 | 52 |
|  | Phenylpropanoid biosynthesis | map00940 | 223 | 199 | 6764 | 9680 | 1.11E-06 | 2.18E-05 | 111 | 112 |
|  | Carbon metabolism | map01200 | 521 | 565 | 6466 | 9314 | 3.87E-06 | 6.67E-05 | 309 | 212 |

**Supplementary Table S2 Continued.**

| **Group** | **Pathway Name** | **Map** | **Count1** | **Count2** | **Count3** | **Count4** | ***p*** | ***q*** | **Up count** | **Down count** |
| --- | --- | --- | --- | --- | --- | --- | --- | --- | --- | --- |
| Leaf/1P | Carotenoid biosynthesis | map00906 | 71 | 43 | 6916 | 9836 | 5.41E-06 | 8.19E-05 | 50 | 21 |
|  | Glyoxylate and dicarboxylate metabolism | map00630 | 225 | 209 | 6762 | 9670 | 5.94E-06 | 8.19E-05 | 172 | 53 |
|  | Plant hormone signal transduction | map04075 | 302 | 306 | 6685 | 9573 | 1.78E-05 | 0.000223 | 115 | 187 |
|  | Folate biosynthesis | map00790 | 97 | 77 | 6890 | 9802 | 9.05E-05 | 0.001041 | 72 | 25 |
|  | Fructose and mannose metabolism | map00051 | 129 | 114 | 6858 | 9765 | 0.000146 | 0.001553 | 81 | 48 |
|  | Alanine, aspartate and glutamate metabolism | map00250 | 132 | 119 | 6855 | 9760 | 0.000212 | 0.002087 | 95 | 37 |
|  | Stilbenoid, diarylheptanoid and gingerol biosynthesis | map00945 | 14 | 3 | 6973 | 9876 | 0.000688 | 0.006328 | 0 | 14 |
|  | Cutin, suberine and wax biosynthesis | map00073 | 43 | 28 | 6944 | 9851 | 0.000869 | 0.007492 | 29 | 14 |
|  | Glycolysis / Gluconeogenesis | map00010 | 239 | 262 | 6748 | 9617 | 0.002286 | 0.018556 | 128 | 111 |
| 1P/2P | Phenylpropanoid biosynthesis | map00940 | 181 | 241 | 3797 | 12647 | 6.35E-19 | 4.38E-17 | 127 | 54 |
|  | Starch and sucrose metabolism | map00500 | 220 | 358 | 3758 | 12530 | 1.74E-15 | 8.01E-14 | 153 | 67 |
|  | Cyanoamino acid metabolism | map00460 | 121 | 151 | 3857 | 12737 | 1.86E-14 | 6.41E-13 | 77 | 44 |
|  | Biosynthesis of amino acids | map01230 | 312 | 704 | 3666 | 12184 | 4.90E-08 | 1.35E-06 | 215 | 97 |
|  | alpha-Linolenic acid metabolism | map00592 | 64 | 83 | 3914 | 12805 | 6.95E-08 | 1.60E-06 | 42 | 22 |
|  | Cutin, suberine and wax biosynthesis | map00073 | 37 | 34 | 3941 | 12854 | 1.76E-07 | 3.47E-06 | 25 | 12 |
|  | Biosynthesis of unsaturated fatty acids | map01040 | 47 | 56 | 3931 | 12832 | 7.18E-07 | 1.24E-05 | 24 | 23 |
|  | Fatty acid elongation | map00062 | 51 | 70 | 3927 | 12818 | 4.54E-06 | 5.70E-05 | 42 | 9 |
|  | Pyruvate metabolism | map00620 | 135 | 270 | 3843 | 12618 | 4.26E-06 | 5.70E-05 | 80 | 55 |
|  | Fatty acid degradation | map00071 | 100 | 188 | 3878 | 12700 | 1.10E-05 | 0.00011 | 51 | 49 |
|  | Fatty acid metabolism | map01212 | 110 | 213 | 3868 | 12675 | 1.11E-05 | 0.00011 | 70 | 40 |
|  | Plant hormone signal transduction | map04075 | 189 | 419 | 3789 | 12469 | 1.01E-05 | 0.00011 | 108 | 81 |
|  | Cysteine and methionine metabolism | map00270 | 106 | 207 | 3872 | 12681 | 2.08E-05 | 0.000192 | 60 | 46 |
|  | Stilbenoid, diarylheptanoid and gingerol biosynthesis | map00945 | 12 | 5 | 3966 | 12883 | 5.35E-05 | 0.000461 | 9 | 3 |
|  | Pentose and glucuronate interconversions | map00040 | 39 | 55 | 3939 | 12833 | 8.75E-05 | 0.00071 | 32 | 7 |

**Supplementary Table S2 Continued.**

| **Group** | **Pathway Name** | **Map** | **Count1** | **Count2** | **Count3** | **Count4** | ***p*** | ***q*** | **Up count** | **Down count** |
| --- | --- | --- | --- | --- | --- | --- | --- | --- | --- | --- |
| 1P/2P | Valine, leucine and isoleucine biosynthesis | map00290 | 52 | 85 | 3926 | 12803 | 0.000112 | 0.000858 | 43 | 9 |
|  | Biotin metabolism | map00780 | 24 | 27 | 3954 | 12861 | 0.000204 | 0.001481 | 20 | 4 |
|  | Pantothenate and CoA biosynthesis | map00770 | 48 | 79 | 3930 | 12809 | 0.000226 | 0.001562 | 28 | 20 |
|  | Monobactam biosynthesis | map00261 | 17 | 16 | 3961 | 12872 | 0.000457 | 0.003006 | 13 | 4 |
|  | Phenylalanine, tyrosine and tryptophan biosynthesis | map00400 | 49 | 85 | 3929 | 12803 | 0.000481 | 0.003019 | 34 | 15 |
|  | Valine, leucine and isoleucine degradation | map00280 | 91 | 192 | 3887 | 12696 | 0.000576 | 0.003457 | 40 | 51 |
|  | 2-Oxocarboxylic acid metabolism | map01210 | 92 | 195 | 3886 | 12693 | 0.000604 | 0.003474 | 64 | 28 |
|  | Phenylalanine metabolism | map00360 | 36 | 57 | 3942 | 12831 | 0.000784 | 0.004328 | 15 | 21 |
|  | Lysine degradation | map00310 | 54 | 101 | 3924 | 12787 | 0.000978 | 0.00519 | 32 | 22 |
|  | Ubiquinone and other terpenoid-quinone biosynthesis | map00130 | 49 | 91 | 3929 | 12797 | 0.001461 | 0.007465 | 42 | 7 |
|  | MAPK signaling pathway - plant | map04016 | 119 | 284 | 3859 | 12604 | 0.00321 | 0.015821 | 79 | 40 |
|  | Fatty acid biosynthesis | map00061 | 57 | 117 | 3921 | 12771 | 0.003583 | 0.016483 | 47 | 10 |
|  | Linoleic acid metabolism | map00591 | 23 | 34 | 3955 | 12854 | 0.00357 | 0.016483 | 20 | 3 |
|  | Butanoate metabolism | map00650 | 33 | 62 | 3945 | 12826 | 0.009088 | 0.040457 | 16 | 17 |
|  | Base excision repair | map03410 | 44 | 91 | 3934 | 12797 | 0.010576 | 0.045609 | 28 | 16 |
| 2P/3P | Protein processing in endoplasmic reticulum | map04141 | 234 | 643 | 3191 | 12798 | 1.81E-06 | 0.00025 | 61 | 173 |

1P, 2P, 3P, and 4P represent one-, two-, three-, and four-year-old pseudobulbs, respectively. N: the number of genes with KEGG pathway annotation in all genes; n: the number of DEGs in N; M: the number of genes annotated to a specific KEGG pathway among all genes; m: the number of DEGs annotated to a specific KEGG pathway. Count1, Count2, Count3, Count4: four data for the Fisher’s test, which are m, M-m, n-m, N-n-M+n, respectively. *p*: *p*-value, *q*: corrected *p*-value. Up and Down count: the number of up-regulated and down-regulated genes annotated to the pathway in the DEGs. *q* = 0.05 as the threshold.

**Supplementary Table S3 Genes related to the biosynthesis of glucomannan**

| **Enzyme name** | **Gene name** | **Enzyme code** | **PFAM Description** | **Gene number** | | |
| --- | --- | --- | --- | --- | --- | --- |
|  |  |  |  | *B. striata* | *Oryza sativa* | *Arabidopsis thaliana* |
| sucrose-proton symporter | *SUC* | - | MFS/sugar transport protein | 17 | 5 | 7 |
| beta-fructofuranosidase | *INV* | 3.2.1.26 | Glycosyl hydrolases family 32 N-terminal domain | 4 | 3 | 6 |
| sucrose synthase | *SUS* | 2.4.1.13 | Sucrose synthase | 6 | 10 | 6 |
| fructokinase | *scrK* | 2.7.1.4 | pfkB family carbohydrate kinase | 13 | 1 | 9 |
| mannose-6-phosphate isomerase | *manA* | 5.3.1.8 | Phosphomannose isomerase type I | 6 | 3 | 2 |
| phosphomannomutase | *PMM* | 5.4.2.8 | haloacid dehalogenase-like hydrolase | 4 | 1 | 1 |
| mannose-1-phosphate guanylyltransferase | *GMPP* | 2.7.7.13 | Nucleotidyl transferase | 5 | 3 | 3 |
| hexokinase | *HK* | 2.7.1.1 | Hexokinase | 13 | 10 | 8 |
| phosphoglucomutase | *pgm* | 5.4.2.2 | Phosphoglucomutase/phosphomannomutase, alpha/beta/alpha domain I | 9 | 3 | 3 |
| UTP--glucose-1-phosphate uridylyltransferase | *UGP2* | 2.7.7.9 | UTP--glucose-1-phosphate uridylyltransferase | 5 | 5 | 5 |
| glucose-6-phosphate isomerase | *GPI* | 5.3.1.9 | Phosphoglucose isomerase | 5 | 4 | 1 |
| cellulose synthase like A | *CSLA* | 2.4.1.32 | Glycosyltransferase like family 2 | 9 | 8 | 9 |

GMPP: mannose-1-phosphate guanylyltransferase or GDP-mannose pyrophosphorylase;

**Supplementary Table S4 Primers used in this study**

| Genes and uses | Forward primer | Reverse primer |
| --- | --- | --- |
| qRT-PCR | | |
| *BsACT1* | GGTCGTGACCTTACTGATGC | CCTCAGGGCACCTGAAACGC |
| *BsUPL1* | GGACCTCACTTTTACCATGG | CTGTGGCCTAATAGCTGTTG |
| *Bs03G11846* | AAGCAGGTGCCCTGAAAGAG | TGGCTGATATTCGCCAGATC |
| *Bs03G11847* | GGTTGTCTTTTGGGTACTGT | CAAGAGGATAAAACCTATCC |
| *Bs03G11848* | TGGGAAATTTTATTTGCC | GCAGTTGGTAGATATATCAT |
| *Bs03G11849* | TGGGAGGTTCTATCGGCA | GTAGTTGGTAGATATATATA |
| *Bs04G13044* | CGCAAACTCCAACGAGTGTCT | CTCGAACAGCCAAGTCCATG |
| *Bs05G15273* | CGTGAATGCGAATGAGTGCT | TATCCTCAACTGTAGTTCGG |
| *Bs07G17879* | GTGCGCAAGATTATAACCCAC | GCCTTCATTCTATGCAAGGAC |
| *Bs07G17880* | CATCACTTCCCCATGGTGCT | CTTCCCAATTTTGTGCCATC |
| *Bs10G03323* | CCGGTTTCAACAACATCGAT | CAGGAATCAGAACAGAGAGTGG |
| Subcellular localization assay (pMDC83-eYFP) | | |
| *SUC* | | |
| *Bs07G17888* | GGGACTAGTATGAGATCGGAGGAGCGAA | CTTACTAGTTCAGTGCATGCCGCCC |
| *Bs08G19590* | CTTACTAGTATGGACGGCGCGGTCT | GGGACTAGTTCAACCAAACCCATGG |
| *INV* | | |
| *Bs02G09995* | CTTACTAGTATGCTTCAATGGCAGCGGAC | GGGACTAGTTCATGTGAATCGGTATGCTTG |
| *Bs04G13083* | GGGACTAGTATGAGAAGTTCTAAGGAAGTT | CTTACTAGTTTACTTGGTTGGGTCGT |
| *Bs06G17791* | GGGACTAGTATGAACATCTATGATCTAGAG | CTTACTAGTTTACAAGGCTCTCATGCGAGG |
| *SUS* | | |
| *Bs09G22402* | CTTACTAGTATGGCGACGCCGGTGC | CTTACTAGTTCTAAACAACCGCAAGT |
| *manA* | | |
| *Bs02G10654* | CTTACTAGTATGGAGGCAGCTGCGGCGGCGG | GGGACTAGTCTAATCGACAAAGAACCGGCTA |
| *CSLA* | | |
| *Bs03G11846* | CTTACTAGTATGGAGGCGAGGGCAT | CTTACTAGTCTAGGAACTGGAGATG |
| *Bs03G11847* | GGGACTAGTATGGAAGCTGCCACC | CTTACTAGTCTAATTGTCCCTTGG |
| *Bs03G11848* | GGGACTAGTATGGAAGGTTTAACT | CTTACTAGTCTAATTGTCCCTTGG |
| *Bs03G11849* | GGGACTAGTATGGAAGGTGCAACTA | CTTACTAGTCTAATTGTCCCTTGGA |
| *Bs04G13044* | CTTACTAGTATGGAGACCGCGACGT | CTTACTAGTTCAGGAGTGAGGGACA |
| *Bs05G15273* | CTTACTAGTATGGAGGGGGCGAGC | GGGACTAGTTTAGGGGTAAGAGAC |
| *Bs07G17879* | GGGACTAGTATGAGGAATGTGTTT | CTTACTAGTCTATGACTTGGAGCC |
| *Bs07G17880* | CTTACTAGTATGCCACTGCAGGCGG | GGGACTAGTTTACTTGTCATTTTGG |
| *Bs10G03323* | CTTACTAGTATGGGAGAGGTTGTGG | GGGACTAGTTCAAGAAATTTTAACAG |
| Virus-mediated gene silencing (pCa-γbLIC) | | |
| *Bs03G11846* | AAGGAAGTTTAAGCGGTCGCCGGAGAC | AACCACCACCACCGTTGTACCGCTTCTCCG |
| *Bs03G11849* | AAGGAAGTTTAAGTATCAACCGATAGGC | AACCACCACCACCGTCAAGCTCCCAAGCTC |
| *Bs04G13044* | AAGGAAGTTTAAAGACCGCGACGTCGT | AACCACCACCACCGTGGACCATGGGGAATG |

**Supplementary Table S4 Continued.**

| Genes and uses | Forward primer | Reverse primer |
| --- | --- | --- |
| DUALmembrane pairwise system yeast two-hybrid (Y2H) | | |
| pBT3-STE | | |
| *Bs03G11846* | CTTGGCCATTACGGCCGAGGCGAGGGCATTGG | CTTGGCCGAGGCGGCCCCGGAACTGGAGATGAAAG |
| *Bs03G11847* | GGGGGCCATTACGGCCGAAGCTGCCACCGTTAC | CTTGGCCGAGGCGGCCCCATTGTCCCTTGGAACT |
| *Bs03G11848* | GGGGGCCATTACGGCCGAAGGTTTAACTAAA | CTTGGCCGAGGCGGCCCCATTGTCCCTTGGAAC |
| *Bs03G11849* | GGGGGCCATTACGGCCGAAGGTGCAACTAACC | CTTGGCCGAGGCGGCCCCATTGTCCCTTGGAACT |
| *Bs04G13044* | CTTGGCCATTACGGCCGAGACCGCGACGTCGT | CTTGGCCGAGGCGGCCCCGGAGTGAGGGACAAAT |
| pPR3-N | | |
| *Bs03G11846* | CTTGGCCATTACGGCCATGGAGGCGAGGGCAT | CTTGGCCGAGGCGGCCCTAGGAACTGGAGATG |
| *Bs03G11847* | GGGGGCCATTACGGCCATGGAAGCTGCCACCG | CTTGGCCGAGGCGGCCCTAATTGTCCCTTGGA |
| *Bs03G11848* | GGGGGCCATTACGGCCATGGAAGGTTTAACT | CTTGGCCGAGGCGGCCCTAATTGTCCCTTGG |
| *Bs03G11849* | GGGGGCCATTACGGCCATGGAAGGTGCAACTA | CTTGGCCGAGGCGGCCCTAATTGTCCCTTGGA |
| *Bs04G13044* | CTTGGCCATTACGGCCATGGAGACCGCGACG | CTTGGCCGAGGCGGCCTCAGGAGTGAGGGAC |
| Luciferase complementation assay (pCCL and JW771) | | |
| *Bs03G11846* | CTTGGTACCATGGAGGCGAGGGCAT | CTTGGTACCGGAACTGGAGATGAAA |
| *Bs03G11847* | CTTGGTACCATGGAAGCTGCCACCG | CTTGTCGACATTGTCCCTTGGAACT |
| *Bs03G11848* | GGGGGTACCATGGAAGGTTTAACT | CTTGTCGACATTGTCCCTTGGAAC |
| *Bs03G11849* | CTTGGTACCATGGAAGGTGCAACTA | CTTGTCGACATTGTCCCTTGGAACT |
| *Bs04G13044* | CTTGGTACCATGGAGACCGCGACGT | CTTGTCGACGGAGTGAGGGACAAAT |
| Arabidopsis |  |  |
| *AtACT2RT* | GGCAAGTCATCACGATTGGT | GTGGTTCCACCACTGAGCAC |
| *LBb1.3* | ATTTTGCCGATTTCGGAAC |  |
| *SALK_065083* | TTCCGTACATAAAATGCAGCC | GCCTCATGCTGGTTACAACTC |
| *SALK_087535* | AGGACACAAACCAAGATCTCG | CAGGATCAGGCTGAAAATCAG |
| *SALK_071916* | CATTTTCACTAGATCCGCCAC | TCCGGTACAAGAACTGTAGCG |

**Supplementary Table S5 Pearson correlation analysis between polysaccharide content and gene expression level.**

| Gene | Gene ID | Pearson correlation coefficients | Significance（Two-tail） |
| --- | --- | --- | --- |
| *BsSUC* | *Bs03G11882* | -0.202 | 0.664 |
|  | *Bs03G12866* | -0.413 | 0.357 |
|  | *Bs07G17888* | 0.195 | 0.676 |
|  | *Bs08G19590* | -0.382 | 0.398 |
|  | *Bs08G19603* | -0.318 | 0.487 |
|  | *Bs08G19604* | -0.144 | 0.758 |
|  | *Bs10G03297* | -0.061 | 0.896 |
|  | *Bs10G03298* | -0.180 | 0.699 |
|  | *Bs13G06055* | -0.067 | 0.887 |
|  | *Bs0023N32790* | -0.340 | 0.456 |
| *BsINV* | *Bs02G09995* | -0.429 | 0.337 |
|  | *Bs04G13083* | -0.352 | 0.438 |
|  | *Bs06G17791* | -0.406 | 0.367 |
|  | *Bs10G02210* | -0.390 | 0.388 |
| *BsSUS* | *Bs01G00350* | -0.168 | 0.720 |
|  | *Bs02G09910* | -0.323 | 0.479 |
|  | *Bs02G09912* | -0.302 | 0.510 |
|  | *Bs03G12144* | -0.054 | 0.909 |
|  | *Bs09G22402* | 0.255 | 0.581 |
|  | *Bs14G06966* | -0.109 | 0.816 |
| *BsscrK* | *Bs02G09889* | 0.193 | 0.678 |
|  | *Bs03G12443* | -0.407 | 0.365 |
|  | *Bs04G12908* | -0.449 | 0.312 |
|  | *Bs04G12909* | 0.264 | 0.567 |
|  | *Bs04G14302* | -0.326 | 0.476 |
|  | *Bs08G20144* | -0.276 | 0.549 |
|  | *Bs08G20145* | -0.412 | 0.358 |
|  | *Bs09G21315* | -0.377 | 0.404 |
|  | *Bs10G02634* | 0.477 | 0.279 |
|  | *Bs11G04045* | -0.371 | 0.413 |
|  | *Bs12G04925* | -0.294 | 0.523 |
| *BsmanA* | *Bs02G10654* | 0.944 | 0.00136 |
| *BsPMM* | *Bs04G13000* | 0.021 | 0.964 |
| *BsGMPP* | *Bs01G00384* | -0.549 | 0.202 |
|  | *Bs03G12806* | -0.016 | 0.972 |
|  | *Bs08G20453* | 0.186 | 0.690 |
|  | *Bs08G20952* | -0.433 | 0.332 |
|  | *Bs13G05751* | -0.418 | 0.350 |

**Supplementary Table S5 Continued.**

| Gene | Gene ID | Pearson correlation coefficients | Significance（Two-tail） |
| --- | --- | --- | --- |
| *BsHK* | *Bs03G11397* | -0.416 | 0.353 |
|  | *Bs04G13160* | 0.628 | 0.131 |
|  | *Bs06G17282* | -0.474 | 0.282 |
|  | *Bs07G19263* | 0.918 | 0.004 |
|  | *Bs10G02464* | 0.787 | 0.036 |
|  | *Bs12G05166* | -0.052 | 0.912 |
| *Bspgm* | *Bs05G14857* | -0.431 | 0.334 |
|  | *Bs09G21940* | -0.345 | 0.449 |
|  | *Bs11G04073* | 0.811 | 0.027 |
|  | *Bs12G04612* | -0.126 | 0.787 |
|  | *Bs16G09437* | 0.549 | 0.202 |
|  | *Bs1025N23032* | -0.198 | 0.672 |
| *Bsugp2* | *Bs02G09921* | -0.186 | 0.690 |
|  | *Bs06G16726* | -0.310 | 0.498 |
|  | *Bs0020N30823* | -0.438 | 0.325 |
| *BsGPI* | *Bs11G03798* | 0.891 | 0.007 |
|  | *Bs12G05424* | -0.131 | 0.780 |
| *BsCSLA* | *Bs03G11846* | -0.078 | 0.867 |
|  | *Bs03G11847* | 0.767 | 0.044 |
|  | *Bs03G11848* | 0.785 | 0.037 |
|  | *Bs03G11849* | 0.754 | 0.050 |
|  | *Bs04G13044* | -0.394 | 0.382 |
|  | *Bs05G15273* | -0.166 | 0.722 |
|  | *Bs07G17879* | 0.249 | 0.591 |
|  | *Bs07G17880* | -0.445 | 0.317 |
|  | *Bs10G03323* | -0.366 | 0.419 |
